# Taphonomic experiments reveal authentic molecular signals for fossil melanins and verify preservation of phaeomelanin in fossils

**DOI:** 10.1038/s41467-023-40570-w

**Published:** 2023-10-06

**Authors:** Tiffany S. Slater, Shosuke Ito, Kazumasa Wakamatsu, Fucheng Zhang, Peter Sjövall, Martin Jarenmark, Johan Lindgren, Maria E. McNamara

**Affiliations:** 1 School of Biological, Earth and Environmental Sciences, University College Cork, Cork, Ireland; 2 Environmental Research Institute, University College Cork, Cork, Ireland; 3 Institute for Melanin Chemistry, Fujita Health University, Toyoake, Aichi Japan; 4 Institute of Geology and Paleontology, Linyi University, Linyi City, Shandong China; 5 RISE Research Institutes of Sweden, Materials and Production, 501 15 Borås, Sweden; 6 Department of Geology, Lund University, 223 62 Lund, Sweden

**Keywords:** Biogeochemistry, Palaeontology

## Abstract

Melanin pigments play a critical role in physiological processes and shaping animal behaviour. Fossil melanin is a unique resource for understanding the functional evolution of melanin but the impact of fossilisation on molecular signatures for eumelanin and, especially, phaeomelanin is not fully understood. Here we present a model for the chemical taphonomy of fossil eumelanin and phaeomelanin based on thermal maturation experiments using feathers from extant birds. Our results reveal which molecular signatures are authentic signals for thermally matured eumelanin and phaeomelanin, which signatures are artefacts derived from the maturation of non-melanin molecules, and how these chemical data are impacted by sample preparation. Our model correctly predicts the molecular composition of eumelanins in diverse vertebrate fossils from the Miocene and Cretaceous and, critically, identifies direct molecular evidence for phaeomelanin in these fossils. This taphonomic framework adds to the geochemical toolbox that underpins reconstructions of melanin evolution and of melanin-based coloration in fossil vertebrates.

## Abstract (web summary)

Reconstructing original fossil colour provides insights into the behaviour of ancient animals but is challenging because phaeomelanin pigments have a poor fossil record. Here, the authors present experimental data that predict the composition of fossil melanins and support the molecular preservation of phaeomelanin in 10 million year old frogs.

## Introduction

Melanin pigments are heterogenous polymers that have critical functions in animal homeostasis^1,2^ and behaviour^3^ and are implicated in key transitions in vertebrate evolution^4^. Integumentary melanins sequester metals and shield tissues from photo-oxidation and mechanical abrasion while providing visual signals that thwart predators and/or attract mates^4,5^. Diverse melanin-based integumentary colours are achieved using eumelanin, which generates black and brown hues, and/or phaeomelanin, which generates rufous and grey colours^6,7^. These pigments are stored in melanosomes—organelles that, in feathers, can have relatively spherical (phaeomelanosomes) to elongate (eumelanosomes) shapes. Evidence of melanosomes and of melanins in the fossil record has fuelled interpretations of the original colours of diverse ancient animals^8–12^, yielding potential insights into behavioural ecology^13–19^ and the functional evolution of melanin^4,20,21^.

Reconstructions of melanin-based animal colour are accurate only where evidence of eumelanin and/or phaeomelanin—or their degraded remains—can be identified with confidence in fossils^22,23^. Early reconstructions attributed specific feather colours to body regions based on melanosomes of different geometries and, presumably, different chemistries^8–11^. More recent research, however, has demonstrated that colour reconstructions based on morphological data alone are only 61.9% accurate^24^. Chemical data, especially on melanin monomers, offer the potential to inform, and enhance the accuracy of, colour models. Such chemical data are especially important for integumentary and non-integumentary tissues that lack a correlation between melanosome shape and chemistry, thus contributing essential data to models of the molecular evolution of melanin pigments. Ideally, chemical evidence of ancient melanin should be supported by morphological evidence of melanosome preservation^23^. Evidence of eumelanin has been recovered from various fossils using time-of-flight secondary ion mass spectrometry (ToF-SIMS), which yields characteristic ion fragmentation patterns for eumelanin^13,15,25–30^ and can map spatial distributions of specific melanin-associated ions onto fossil melanosomes^15,26^. Evidence of eumelanin in fossils can also be inferred from associations between preserved melanosomes and specific metal species, e.g., Cu^2+^, and/or metal coordination complexes^31^. Neither of these methods, however, can confirm the preservation of eumelanin molecular units (i.e., monomers) in fossils.

Chemical evidence of fossil phaeomelanin is even more elusive. The ornithischian dinosaur *Borealopelta* preserves evidence for the phaeomelanin subunit benzothiazole (BZ) in ToF-SIMS spectra but lacks preserved melanosomes^16^; whether the BZ signal is original, or a diagenetic or anthropogenic contaminant, has not been conclusively demonstrated. Evidence of BZ has also been reported in the hair of the three million year old fossil mouse *Apodemus* based on synchrotron-S-XANES spectra and Zn-organosulfur associations^12^. Melanosomes are preserved in the fossil hair, but detailed molecular chemistry (e.g., alkaline hydrogen peroxide oxidation followed by high-performance liquid chromatography (AHPO-HPLC) data on melanin monomers) was not reported. Elevated concentrations of S and/or organosulfur compounds in fossil melanosomes have also been interpreted as evidence of phaeomelanin^27^, but this could reflect chemical alteration of eumelanin during fossilisation, e.g., via sulfurisation^29^. A phaeomelanin-dominated composition has been inferred for putative melanosomes from two bats from the 47 Ma Messel biota based on their small and subrounded geometries^27^. The chemistry of the structures (as reflected in the plotted position of the fossils in the principal components chemospace), however, is not consistent with enrichment in sulfur-bearing molecular fragments^27^.

Identifying authentic signals for fossil melanins, and discriminating these from the effects of fossilisation, is therefore essential to understanding the fossil record of melanin. The pathways by which fossilisation can impact chemical evidence of melanin, however, are not fully understood. Taphonomic experiments on melanosomes have yielded insights into how fossilisation processes can alter melanosome morphology and geochemistry^27,32,33^. Recent experiments on synthetic melanins^25^ suggested that different eumelanin subunits converge in chemistry during thermal maturation. It is unclear, however, whether the latter changes also apply to natural melanins, especially in the complex chemical environment represented by intact melanosomes and tissues.

**Fig. 1:**
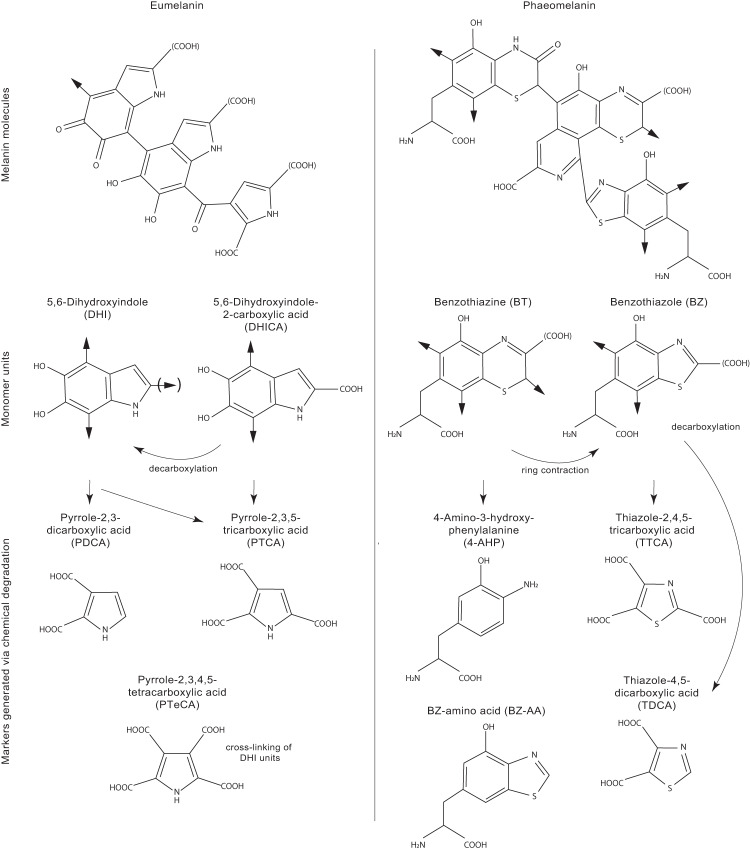
Eumelanin and phaeomelanin molecules and their respective constituent monomers. Key eumelanin monomer units are 5,6-dihydroxyindole (DHI) and 5,6-dihydroxyindole-2-carboxylic acid (DHICA); phaeomelanin monomer units are benzothiazine (BT) and benzothiazole (BZ). Chemical degradation of these units by alkaline hydrogen peroxide oxidation (AHPO) generates PDCA (pyrrole-2,3-dicarboxylic acid), PTCA (pyrrole-2,3,5-tricarboxylic acid), PTeCA (pyrrole-2,3,4,5-tetracarboxylic acid), TTCA (thiazole-2,4,5-tricarboxylic acid) and TDCA (thiazole-4,5-dicarboxylic acid) markers. Hydroiodic acid (HI) hydrolysis generates 4-AHP (4-amino-3-hydroxyphenylalanine), BZ-AA (benzothiazole amino acid) and 3-AHP (3-amino-4-hydroxyphenylalanine; see Supplementary Fig. 16)^33–36,40,41^.

The most diagnostic chemical assay for eumelanin is AHPO-HPLC^34^. This assay is the accepted standard in the field of melanin biochemistry for the analysis of the molecular composition of melanin^35^ and quantifies derivatives of discrete eumelanin monomers (Fig. 1)^36,37^. The approach has revealed molecular preservation of eumelanin in fossil squid^34,38^, insects^39^, an ichthyosaur^40^ and a frog^20^. Phaeomelanin monomers can be identified using both AHPO-HPLC and HPLC following HI hydrolysis of samples^41^, but these approaches have not been applied to the detection of phaeomelanin in fossils.

Here we use taphonomic experiments on feathers from extant birds and AHPO-HPLC analysis to examine the impact of thermal maturation on eumelanin and phaeomelanin, including their respective monomers, in a whole-tissue context. Our results reveal which molecular signatures generated by AHPO-HPLC are authentic signals for thermally matured melanin. Our empirical model is tested using data from the nonavian dinosaur *Sinornithosaurus*, the fossil bird *Confuciusornis* and specimens of the fossil frog *Pelophylax pueyoi* (Supplementary Fig. 1). Critically, our experimental data support evidence for the preservation of phaeomelanin in all fossils analysed. Our predictive model of eumelanin and phaeomelanin degradation in whole tissues thus provides a taphonomic framework for the identification of eumelanin and phaeomelanin monomers in fossils that will underpin future investigations of the evolution of melanin and its functions through deep time.

## Results and discussion

### Melanin chemistry of untreated feathers

Eumelanin comprises 5,6-dihydroxyindole (DHI) and 5,6-dihydroxyindole-2-carboxylic acid (DHICA) units^36,37^ (Fig. 1). AHPO-HPLC of DHI yields primarily pyrrole-2,3-dicarboxylic acid (PDCA) and pyrrole-2,3,4,5-tetracarboxylic acid (PTeCA) cross-linked at the C2 and C3 positions^25,37^; minor pyrrole-2,3,5-tricarboxylic acid (PTCA) reflects cross-linking at the C2 position^36,37^. AHPO-HPLC of DHICA yields primarily PTCA^36,37^ and PTeCA cross-linked at the C3 position^25,37^. Phaeomelanin comprises benzothiazine (BT) and BZ units (Fig. 1), the latter generating thiazole-2,4,5-tricarboxylic acid (TTCA) and, if decarboxylated, thiazole-4,5-dicarboxylic acid (TDCA)^36^. Hydroiodic acid (HI) hydrolysis of phaeomelanin allows direct identification of the BZ monomer 6-(2-amino-2carboxyethyl)-4-hydroxybenzothiazole (BZ-amino acid, BZ-AA) and the degradation products of BT, 4-amino-3-hydroxyphenylalanine (4-AHP) and 3-amino-4-hydroxyphenylalanine (3-AHP)^41,42^. 3-AHP is a minor degradation product of phaeomelanin but is not diagnostic^41^.

Feathers comprise largely non-melanin proteins such as corneous beta-proteins (formerly termed beta-keratins^43^). Degradation products of these proteins, however, are highly unlikely to be confused with those of melanins in AHPO-HPLC data (see Methods).

**Fig. 2:**
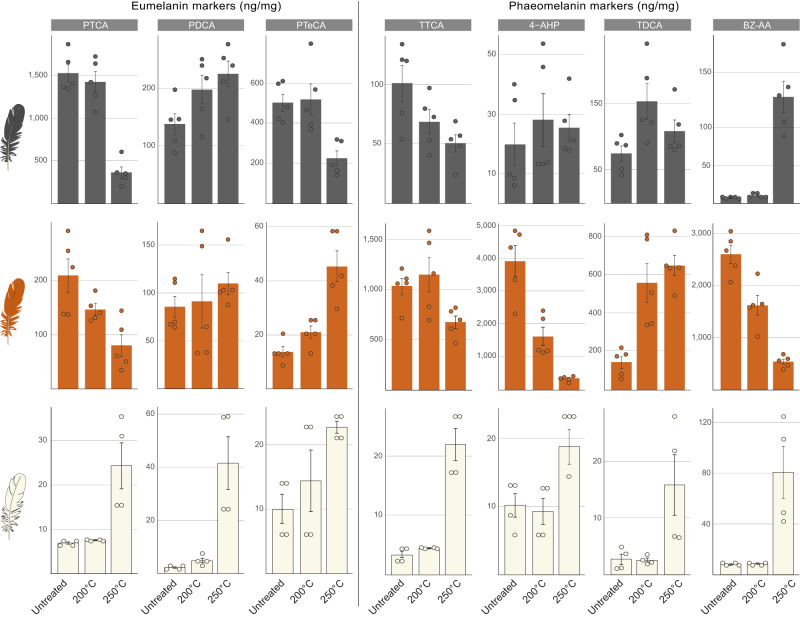
AHPO-HPLC data for black, rufous and white feathers from extant birds with, and without, thermal treatment. Bar charts show melanin marker concentrations in black and rufous (each *n* = 5; *Gallus gallus*) and white (n = 4; *Egretta garzetta*) feathers; data are shown for untreated feathers and feathers thermally matured at 200 °C and 250 °C. Melanin markers are generated during alkaline hydrogen peroxide oxidation (AHPO) and hydroiodic (HI) acid hydrolysis. Data are presented as mean ± SD. PTCA pyrrole-2,3,5-tricarboxylic acid, PDCA pyrrole-2,3-dicarboxylic acid, PTeCA pyrrole-2,3,4,5-tetracarboxylic acid, TTCA thiazole-2,4,5-tricarboxylic acid, 4-AHP 4-amino-3-hydroxyphenylalanine, TDCA thiazole-4,5-dicarboxylic acid, BZ-AA benzothiazole amino acid. Source data are provided as a Source Data file.

Soluene-350 solubilisation of untreated black feathers reveals a mean eumelanin: total melanin ratio (A650: A500 = 0.30 ± 0.01) that is consistent with a predominantly eumelanic composition^44^. In contrast, untreated rufous feathers show a mean eumelanin: total melanin ratio (A650: A500 = 0.097 ± 0.00) that indicates a primarily phaeomelanic composition^44^. These spectrophotometric data are supported by the results of AHPO-HPLC analyses. As expected, our AHPO-HPLC data reveal that black and rufous feathers are dominated by eumelanin and phaeomelanin markers, respectively (Fig. 2, Table 1 and Supplementary Fig. 2). Phaeomelanin markers also occur in black feathers, and eumelanin markers in rufous feathers, albeit as minor components in each case.

**Table 1:** AHPO-HPLC data for total melanin marker concentrations (ng/mg) in black, rufous and white feathers

Feather type		HCl pretreatment	Total melanin	Eumelanin	Phaeomelanin
Black	Untreated	no	2377 ± 309	2173 ± 315	204 ± 29
		yes	1059 ± 170	988 ± 143	71 ± 28
	200 °C	no	2408 ± 474	2146 ± 410	261 ± 89
	250 °C	no	1144 ± 249	819 ± 215	325 ± 53
		yes	786 ± 204	702 ± 179	83 ± 28
Rufous	Untreated	no	7984 ± 1411	308 ± 67	7676 ± 1356
		yes	583 ± 60	84 ± 16	499 ± 54
	200 °C	no	5178 ± 1041	258 ± 54	4920 ± 1005
	250 °C	no	2412 ± 379	235 ± 40	2177 ± 381
		yes	1263 ± 91	195 ± 29	1068 ± 68
White	Untreated	no	43 ± 7	20 ± 2	24 ± 5
		yes	21 ± 4	8 ± 0	13 ± 4
	200 °C	no	51 ± 13	22 ± 4	29 ± 10
	250 °C	no	226 ± 19	85 ± 31	141 ± 48
		yes	173 ± 36	152 ± 39	22 ± 4
Percentage Change In Marker Concentration Relative To Untreated Samples					
Black	200 °C	no	+1	−1	+28
	250 °C	no	−52	−62	+59
		yes	−26	−28	+18
Rufous	200 °C	no	−35	−16	−36
	250 °C	no	−69	−24	−72
		yes	+116	+132	+114
White	200 °C	no	+18	+12	+22
	250 °C	no	+420	+333	+491
		yes	+712	+1807	+63

Mean and standard deviation data (in ng/mg) for concentrations of total melanin, total eumelanin (PTCA + PDCA + PTeCA) and total phaeomelanin (TTCA + TDCA + 4-AHP + BZ-AA) markers obtained using alkaline hydrogen peroxide oxidation (AHPO) and hydroiodic (HI) acid hydrolysis. Black and rufous feathers, *Gallus gallus*; white feathers, *Egretta garzetta*. Source data are provided as a Source Data file.

Untreated black feathers contain 2377 ng/mg total melanin markers, which are predominantly eumelanin markers (91%: 2173 ng/mg; Table 1), in turn dominated by PTCA (1531 ng/mg), with minor PTeCA (505 ng/mg) and PDCA (138 ng/mg; Fig. 2, Table 2 and Supplementary Fig. 2). The PDCA: PTCA ratio is 0.090, indicating that eumelanin in the black feather comprises ca. 66% DHI and 34% DHICA^45^. The PTeCA: PTCA ratio is 0.33. The accessory phaeomelanin signal in the black feathers is dominated by TTCA and TDCA with minor 4-AHP and BZ-AA (Fig. 2, Table 2 and Supplementary Fig. 2).

**Table 2:** AHPO-HPLC data for individual melanin marker concentrations (ng/mg) in black, rufous and white feathers

			Eumelanin markers				Phaeomelanin markers					
Feather type		HCl pre-treatment	PTCA	PDCA	PTeCA	PTeCA: PTCA	TTCA		TDCA	TTCA: TDCA	BZ-AA	4-AHP
Black	Untreated	no	1531 ± 203	138 ± 38	505 ± 87	0.33 ± 0.03	101 ± 31		75 ± 23	1.6 ± 0.96	8 ± 0.93	20 ± 14
		yes	463 ± 111	83 ± 21	443 ± 79	1.01 ± 0.31	17 ± 5		54 ± 24	0.34 ± 0.08	X	X
	200 °C	no	1427 ± 239	198 ± 50	521 ± 156	0.36 ± 0.06	69 ± 20		154 ± 54	0.46 ± 0.09	11 ± 2	28 ± 18
	250 °C	no	367 ± 133	226 ± 45	226 ± 75	0.66 ± 0.22	51 ± 15		109 ± 34	0.51 ± 0.22	140 ± 42	25 ± 9
		yes	289 ± 85	108 ± 22	305 ± 77	1.07 ± 0.09	19 ± 7		64 ± 22	0.31 ± 0.1	X	X
Rufous	Untreated	no	209 ± 62	86 ± 22	14 ± 4	0.07 ± 0.04	1032 ± 169		139 ± 63	9.2 ± 4	2598 ± 346	3907 ± 961
		yes	59 ± 11	16 ± 5	9 ± 4	0.14 ± 0.05	356 ± 33		143 ± 27	2.6 ± 0.5	X	X
	200 °C	no	146 ± 20	91 ± 55	21 ± 5	0.14 ± 0.02	1149 ± 350		555 ± 206	2.12 ± 0.22	1613 ± 383	1603 ± 553
	250 °C	no	80 ± 41	110 ± 24	45 ± 11	0.70 ± 0.3	673 ± 128		647 ± 108	1.03 ± 0.11	384 ± 102	185 ± 74
		yes	100 ± 20	39 ± 11	56 ± 9	0.59 ± 0.17	612 ± 80		456 ± 77	1.4 ± 0.4	X	X
White	Untreated	no	7 ± 1	2 ± 1	10 ± 3	1.5 ± 1	10 ± 4		3 ± 1	3.89 ± 2.47	8 ± 1	3 ± 2
		yes	3 ± 1	2 ± 0	3 ± 0	1.13 ± 0.33	8 ± 3		5 ± 1	1.59 ± 0.19	X	X
	200 °C	no	8 ± 0	5 ± 2	9 ± 3	1.22 ± 0.43	14 ± 8		4 ± 0	3.23 ± 1.81	8 ± 1	2 ± 1
	250 °C	no	24 ± 9	42 ± 17	19 ± 4	0.82 ± 0.12	23 ± 2		22 ± 5	1.09 ± 0.31	81 ± 36	16 ± 9
		yes	73 ± 22	36 ± 6	43 ± 15	0.60 ± 0.11	11 ± 1		11 ± 5	1.24 ± 0.60	X	X
Percentage change in marker concentration relative to untreated samples												
Black	200 °C	no	−7	+44	+3	+10	−32		+105	−71	+34	+41
	250 °C	no	−76	+64	-55	+99	−50		+45	−69	+1620	+28
		yes	−37	+31	-31	+5	+14		+19	−10	X	X
Rufous	200 °C	no	−30	−46	+53	+94	+11		+299	−77	−38	−59
	250 °C	no	−62	+29	+231	+855	−35		+365	−89	−85	−95
		yes	+69	+140	+530	+311	+72		+219	−45	X	X
White	200 °C	no	+9	+108	-9	−20	+43		+35	−17	0	-6
	250 °C	no	+251	+1595	+85	−46	+125		+575	−72	+914	+501
		yes	+2328	+1934	+1250	−47	+30		+117	−22	X	X

Mean and standard deviation data for individual melanin markers in untreated and experimentally matured black and rufous (each *n* = 5; *Gallus gallus*) and white (*n* = 4; *Egretta garzetta*) feathers following alkaline hydrogen peroxide oxidation (AHPO) and hydroiodic (HI) acid hydrolysis. Percentage data denote the mean change in concentrations of specific markers between untreated feathers and those matured at 200 °C and 250 °C. *PTCA* pyrrole-2,3,5-tricarboxylic acid, *PDCA* pyrrole-2,3-dicarboxylic acid, *PTeCA* pyrrole-2,3,4,5-tetracarboxylic acid, *TTCA* thiazole-2,4,5-tricarboxylic acid, *TDCA* thiazole-4,5-dicarboxylic acid, *BZ-AA* benzothiazole amino acid, *4-AHP* 4-amino-3-hydroxyphenylalanine. Source data are provided as a Source Data file.

Total melanin markers are more abundant in untreated rufous feathers (7984 ng/mg) than in black feathers (Table 1). Most markers (96%) are derived from phaeomelanin (7676 ng/mg), predominantly 4-AHP (3907 ng/mg), BZ-AA (2598 ng/mg) and TTCA (1032 ng/mg), with minor TDCA (139 ng/mg; Fig. 2, Table 2 and Supplementary Fig. 2). This composition indicates that phaeomelanin in the rufous feathers comprises approximately equal quantities of BT and BZ units (the conversion factors of 34 for TTCA and 9 for 4-AHP yield estimated quantities of 35163 ng/mg BT and 35088 ng/mg BZ, respectively^36,42^). The accessory eumelanin signal is dominated by PTCA with minor PDCA and PTeCA (Fig. 2, Table 2 and Supplementary Fig. 2).

Untreated white feathers show total melanin concentrations of only 43 ng/mg (Table 1). The dominant markers are TTCA (10 ng/mg) and PTeCA (10 ng/mg); all other markers are present in negligible quantities that approach detection limits (Fig. 2, Table 2 and Supplementary Fig. 2).

### Maturation of black feathers

Following maturation at 200 °C for 1 h (herein termed moderate maturation), concentrations of total melanin markers and eumelanin markers are almost identical to untreated feathers (total melanin: 2408 ng/mg, +1% (relative to untreated); eumelanin: 2146 ng/mg, -1%; Table 1). As with untreated samples, eumelanin markers are dominated by PTCA (1427 ng/mg, -7%; Fig. 2, Table 2 and Supplementary Fig. 2). PTeCA (521 ng/mg, +3%) and PDCA (198 ng/mg, +44%) are minor components and the associated PTeCA: PTCA ratio is 0.36. The minor decrease in PTCA and increase in PDCA likely reflect limited decarboxylation of DHICA, giving rise to DHI (that, in turn, yields primarily PDCA during AHPO-HPLC analysis; Fig. 1)^25,37^. The accessory phaeomelanin signal is dominated by TDCA (Fig. 2, Table 2 and Supplementary Fig. 2).

In contrast, maturation at 250 °C for 1 h (i.e., strong maturation) results in a dramatic loss of markers for total melanin and for eumelanin (total melanin: 1144 ng/mg, -52%; eumelanin: 819 ng/mg, -62%; Table 1). Further, the various eumelanin markers differ in their response to treatment: PTCA and PTeCA decrease (PTCA: 367 ng/mg, -76%; PTeCA: 226 ng/mg, -55%), whereas PDCA increases (226 ng/mg, +64%; Fig. 2, Table 2 and Supplementary Fig. 2). The changes in PDCA and PTCA (Fig. 1) likely reflect extensive decarboxylation of DHICA (resulting in the loss of PTCA and increase in PDCA), yielding abundant non-cross-linked DHI^37^. The decrease in concentration of PTeCA is less than that for PTCA and reflects less extensive thermal degradation of cross-linked DHI and/or DHICA units; the associated increase of the PTeCA: PTCA ratio (to 0.61) indicates selective preservation of cross-linked DHI units^34,37,38^.

Following strong maturation, the accessory phaeomelanin signal in black feathers is dominated by BZ-AA and TDCA. TTCA and 4-AHP are minor components (Fig. 2, Table 2 and Supplementary Fig. 2).

### Maturation of rufous feathers

After moderate maturation, concentrations of total melanin and phaeomelanin markers decrease markedly (total melanin: 5178 ng/mg, -35%; phaeomelanin: 4920 ng/mg, -36%; Table 1). Phaeomelanin markers are dominated by BZ-AA (1613 ng/mg, -38%) and 4-AHP (1603 ng/mg, -59%); TTCA (1149 ng/mg, +11%) and TDCA (555 ng/mg, +299%) are present in minor amounts (Fig. 2, Table 2 and Supplementary Fig. 2). The increase in TDCA during maturation likely reflects progressive decarboxylation of BZ (Fig. 1). The loss of 4-AHP is probably due to the conversion of BT to BZ via ring contraction^42^; decarboxylation of BZ is likely to further contribute to the increase in TDCA. As in untreated feathers, the accessory eumelanin signal is dominated by PTCA (Fig. 2, Table 2 and Supplementary Fig. 2).

Following strong maturation, concentrations of total melanin and phaeomelanin markers decrease further (total melanin: 2412 ng/mg, -69%; phaeomelanin: 2177 ng/mg, -72%; Table 1). Markers are dominated by TTCA (673 ng/mg, -35%) and TDCA (647 ng/mg, +365%; Fig. 2, Table 2 and Supplementary Fig. 2). BZ-AA (384 ng/mg, -85%) and 4-AHP (185 ng/mg, -95%) are present in minor quantities. As with moderately matured samples, the increase in concentrations of TDCA likely reflect the decarboxylation of BZ; the continued loss of 4-AHP probably reflects the conversion of BT to BZ^41^.

Following strong maturation, the accessory eumelanin signal in rufous feathers is dominated by PDCA. PTCA and PTeCA are present in minor amounts; PTCA exhibits a marked decrease, and PDCA a marked increase, compared to untreated feathers (Fig. 2, Table 2 and Supplementary Fig. 2).

### Maturation of white feathers

Moderately matured white feathers show a minor increase in concentrations of total melanin (51 ng/mg, +18%), eumelanin (22 ng/mg, +12%) and phaeomelanin (29 ng/mg, +22%) markers (Table 1); except for TTCA (14 ng/mg), all markers show concentrations <9 ng/mg (Fig. 2, Table 2 and Supplementary Fig. 2). In contrast, strongly matured white feathers show substantial increases in total melanin (226 ng/mg, +420%), eumelanin (85 ng/mg, +333%) and especially phaeomelanin (141 ng/mg, +491%) markers (Table 1). The most abundant marker following maturation at 250 °C is BZ-AA (81 ng/mg, +914%), with minor PDCA (42 ng/mg, +1595%), PTCA (24 ng/mg, +251%), TTCA (23 ng/mg, +125%), TDCA (22 ng/mg, +575%), PTeCA (19 ng/mg, +85%) and 4-AHP (16 ng/mg, +501%; Fig. 2, Table 2 and Supplementary Fig. 2).

The white feathers represent a melanin-poor, protein-rich system. The dramatic increase in concentration of most melanin markers in these samples with progressive maturation is inconsistent with our experimental results for the degradation of eumelanin in eumelanin-dominated systems and of phaeomelanin in phaeomelanin-dominated systems. The chemical changes thus cannot be explained by the degradation of specific melanin monomers. Instead, the white feather data strongly suggest that limited quantities of melanin markers are generated from non-melanin feather components during thermal maturation. This process is enhanced at relatively elevated temperatures, but total marker concentrations remain very low relative to those of black and rufous feathers. Artificial melanin markers in AHPO-HPLC data most likely derive from the degradation of non-melanin proteins^46^ (which comprise ~99% of feathers^47^). We cannot, however, exclude potential contributions from other sources for which an AHPO-HPLC signature is unknown, e.g., products from lipoxidation, glycoxidation^48,49^ and/or the degradation of any non-melanin pigments that may be present in very small quantities.

### Comparative changes in melanin marker abundance among feather types

Overall, eumelanin and phaeomelanin degrade at broadly similar rates during maturation, albeit with a slight bias towards the survival of eumelanin (during strong maturation, concentrations of total markers decrease by 62% for eumelanin and by 72% for phaeomelanin; Table 1). These data indicate that phaeomelanin markers should be detectable (in at least certain fossils) using AHPO-HPLC.

Individual melanin markers lack a consistent response to maturation in black and rufous feathers. PTCA (and thus DHICA) dominates the composition of all black feathers analysed (regardless of experimental treatment) and is the most abundant eumelanin marker in untreated and moderately matured rufous feathers (Fig. 2 and Table 2). In contrast, concentrations of PTCA decrease steadily with progressive maturation in rufous feathers (200 °C: -30%; 250 °C: -62%) but decrease markedly in black feathers only upon maturation at 250 °C (200 °C: -7%; 250 °C: -76%).

The eumelanin marker PDCA is present in low quantities in all feathers analysed, but is the only eumelanin marker to increase substantially in concentration with progressive maturation in black feathers and is the most abundant eumelanin marker in strongly matured rufous and white feathers (Fig. 2 and Table 2). Unexpectedly, PDCA concentrations increase during moderate maturation in black feathers and decrease in rufous feathers (+44% and -46%, respectively), but increase in both feather types during strong maturation (rufous: +29%; black: +64%). The enhanced loss of both PDCA and PTCA in moderately matured rufous feathers (relative to black feathers) cannot be explained readily using the eumelanin-rich feather data (and well-established chemical mechanisms for degradation of eumelanin monomers^25,34–38^) as a model. Instead, these data indicate a heterogeneous response of DHI and DHICA units to thermal maturation in feathers of different colour. This could reflect differences in the extent to which DHI and DHICA units are stabilised by cross-linking, both within the monomers themselves and with other moieties in the local chemical environment, e.g., melanoproteins, proteins, lipids, sugars and/or metals^31^, in feathers of different colour.

PTeCA concentrations decrease with strong maturation in black feathers but increase with both moderate and strong maturation in rufous feathers (Fig. 2 and Table 2). The associated increase in PTeCA: PTCA in rufous feathers (200 °C: +94%; 250 °C: +855%) far exceeds that in black feathers (200 °C: +10%; 250 °C: +99%). This could reflect more cross-linking of eumelanin in rufous relative to black feathers^34,37,38^ or generation of PTeCA via thermal degradation of melanoproteins or other molecules specific to phaeomelanin-dominated systems.

Phaeomelanin markers also exhibit different trends during maturation in rufous and black feathers. In rufous feathers, TTCA decreases in concentration after strong maturation but is the most abundant phaeomelanin marker. TDCA is the only phaeomelanin marker to increase in concentration with progressive maturation (Fig. 2 and Table 2). In contrast, similar trends are not observed for TDCA and TTCA in black feathers with progressive maturation (TTCA: -32% at 200 °C and -50% at 250 °C; TDCA: +105% at 200 °C and +45% at 250 °C). These changes in phaeomelanin marker concentrations in black feathers cannot readily be explained using the phaeomelanin-rich feather data as a model. As with the eumelanin markers discussed above, the heterogeneous response of phaeomelanin markers to maturation may reflect differences in local chemical environment between eumelanin- and phaeomelanin-dominated systems.

4-AHP and BZ-AA are the most abundant phaeomelanin markers in untreated and moderately matured rufous feathers (Fig. 2, Table 2 and Supplementary Fig. 2). During maturation, concentrations of 4-AHP progressively decrease in rufous feathers (200 °C: -59%; 250 °C: -95%) but increase in black feathers (200 °C: +41%; 250 °C: +28%). BZ-AA concentrations decrease progressively with maturation in rufous feathers (200 °C: -38%; 250 °C: -85%). Black feathers show a similar increase in this monomer following moderate maturation (+34%) but a dramatic increase during strong maturation (+1620%). This may reflect, in part, natural variation in a minor component of the feather melanin chemistry. As with TTCA and TDCA, these differences in the response of 4-AHP and BZ-AA in black and rufous feathers are difficult to explain using the well-established chemical mechanisms outlined above^41^ for degradation of phaeomelanin. Instead, the increase in 4-AHP and BZ-AA in black feathers likely reflects minor generation of these moieties from non-melanin feather components, i.e., melanoproteins, proteins, lipids or sugars.

Melanin markers in white feathers are present in sufficiently high concentrations to facilitate comparisons with other feather types only for strongly matured samples (Fig. 2 and Table 2). The dominant trend in the data is for substantial increase in the concentrations of all markers. Given the low abundance of melanin markers in untreated white feathers, the only plausible explanation is that the increase in eumelanin and phaeomelanin markers reflects generation from non-melanin moieties in the feathers during thermal degradation.

The progressive decrease in PTCA with increasing maturation observed in black feathers here is supported by recent maturation experiments using synthetic melanin^25^. There are some differences between the results of the latter experiments and our study, e.g., maturation of synthetic melanins did not yield a progressive increase in PDCA^25^. Here, the increase in PDCA is attributed to DHICA degradation (generating DHI and, upon AHPO-HPLC analysis, PDCA; Fig. 1)^37^. Overall, our data on matured black feathers more closely resemble previously reported data for experimentally matured *Sepia* melanin than those for isolated synthetic melanin units and mixtures thereof^25^. The ratio of phaeomelanin in black feathers increases with strong maturation (untreated: +9%; 250 °C: +28%), due primarily to the dramatic increase in BZ-AA (which likely reflects degradation of non-melanin feather components, e.g., proteins).

Collectively, our data indicate that both eumelanin and phaeomelanin follow distinct chemical pathways during thermal maturation, at least when hosted within tissues.

### HCl-AHPO-HPLC data

HCl treatment prior to AHPO-HPLC is commonly applied^39,40^ to fossil tissue samples to remove associated mineral components; the treatment has also been applied to biological samples to remove potentially interfering compounds such as proteins^46^. The potential impact of acid treatment on AHPO-HPLC data from fossils is, however, unknown. We therefore applied this method to a duplicate set of experimental samples.

**Fig. 3:**
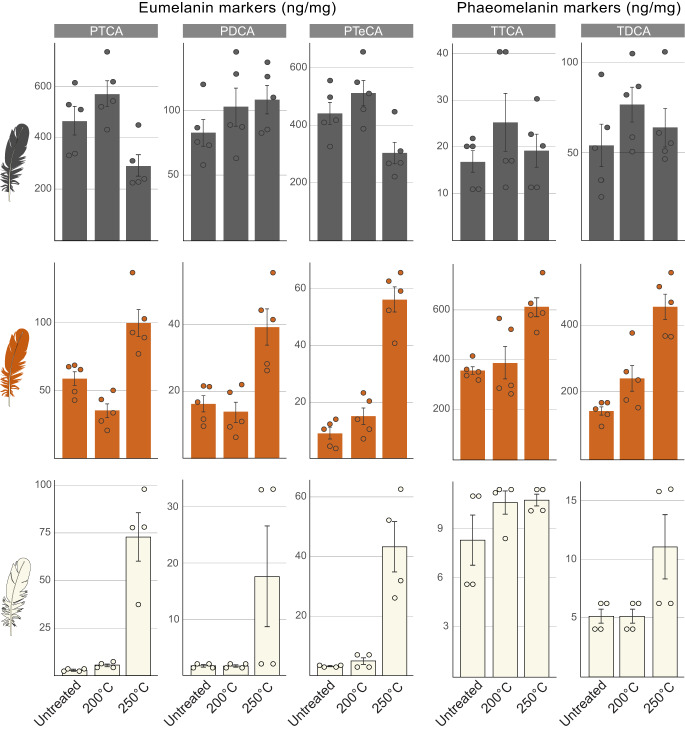
HCl-AHPO-HPLC data for black, rufous and white feathers from extant birds with, and without, thermal treatment. Bar charts show melanin marker concentrations for untreated feathers and feathers thermally matured at 200 °C and 250 °C. Melanin markers are generated during HCl-alkaline hydrogen peroxide oxidation (AHPO). Filled bars indicate the mean values per treatment; floating bars indicate standard deviation per treatment. Black and rufous feathers are from the domestic chicken (each *n* = 5; *Gallus gallus*) and white feathers are from the little egret (*n* = 4; *Egretta garzetta*). Data are presented as mean ± SD. PTCA pyrrole-2,3,5-tricarboxylic acid, PDCA pyrrole-2,3-dicarboxylic acid, PTeCA pyrrole-2,3,4,5-tetracarboxylic acid, TTCA thiazole-2,4,5-tricarboxylic acid, TDCA thiazole-4,5-dicarboxylic acid. Source data are provided as a Source Data file.

Following HCl treatment, unmatured black feathers contain 1059 ng/mg total melanin markers (Table 1). Eumelanin markers dominate (988 ng/mg) with abundant PTCA (463 ng/mg) and PTeCA (443 ng/mg) and minor PDCA (83 ng/mg; Fig. 3, Tables 1, 2 and Supplementary Fig. 3). The PTeCA: PTCA ratio is 1.01 and the accessory phaeomelanin signal is dominated by TDCA (Fig. 3, Table 2 and Supplementary Fig. 3). HCl-treated, unmatured rufous feathers contain fewer melanin markers (583 ng/mg) than black feathers. Phaeomelanin markers dominate (499 ng/mg), especially TTCA (356 ng/mg); TDCA is a minor component (143 ng/mg). Untreated white feathers possess very few melanin markers (total 21 ng/mg), which are dominated by TTCA (8 ng/mg) and TDCA (5 ng/mg).

Following moderate maturation, HCl-treated feathers show broadly similar total, and relative, concentrations of melanin markers to untreated feathers (Fig. 3, Table 2 and Supplementary Fig. 3). Following strong maturation, HCl-treated black feathers contain markedly fewer melanin and eumelanin markers relative to HCl-treated unmatured samples (total melanin: 786 ng/mg, -26%; eumelanin: 702 ng/mg, -28%; Table 1). Eumelanin markers are dominated by PTeCA (305 ng/mg, -31%) and PTCA (289 ng/mg, -37%) with minor PDCA (108 ng/mg, +31%; Fig. 3, Table 2 and Supplementary Fig. 3). TDCA dominates the accessory phaeomelanin signal (Fig. 3, Table 2 and Supplementary Fig. 3).

Strongly matured, HCl-treated rufous feathers contain more melanin markers than black feathers (total melanin: 1263 ng/mg, +116%; phaeomelanin: 1068 ng/mg, +114%; Table 1). Unlike black feathers, phaeomelanin markers show higher concentrations of TTCA (612 ng/mg, +72%) than TDCA (456 ng/mg, +219%). The accessory eumelanin signal is dominated by PTCA with minor PTeCA and PDCA (Fig. 3, Table 2 and Supplementary Fig. 3).

Strongly matured, HCl-treated white feathers contain substantially more total melanin and, especially, eumelanin markers (total melanin: 173 ng/mg, +712%; eumelanin: 152 ng/mg, +1807%); PTCA (73 ng/mg, +2328%), PTeCA (43 ng/mg, +1250%) and PDCA (36 ng/mg, +1934%) concentrations are particularly high (Fig. 3, Table 2 and Supplementary Fig. 3). Concentrations of all other markers are relatively unchanged.

These experimental data reveal important differences in the response of melanins to thermal maturation following, and without, acid treatment. First, fewer melanin markers are recovered following acid treatment (-31%, -48% and -23% for strongly matured black, rufous and white feathers, respectively). This may reflect degradation of melanin markers during acid treatment. Secondly, the relative proportions of the various markers differ markedly between acid-treated and non-acid-treated samples. HCl-treated, strongly matured black feathers retain more PTeCA than samples lacking acid treatment. HCl-treated rufous feathers contain less TTCA, and especially TDCA, than samples lacking acid treatment. HCl-treated, strongly matured white feathers contain more PTeCA, and especially PTCA, relative to samples lacking acid treatment, but total melanin concentrations remain low. This probably reflects the generation of minor amounts of melanin monomers from non-melanin components.

### ToF-SIMS data

ToF-SIMS analyses were conducted on untreated and strongly matured feathers and on melanin extracts from these samples. The spectra of untreated and strongly matured feathers do not show distinct fragment ion patterns for melanin: as in data of synthetic eumelanin and phaeomelanin, *Sepia*melanin and a melanin extract of a rufous zebra finch (*Taeniopygia guttata*) feather (see Supplementary Fig. 4) instead, spectra are dominated by typical fragment ions for protein (probably derived from feather corneous beta-proteins) and lipids (probably derived from feather waxes; Supplementary Figs. 5–9). This likely reflects the dispersed distribution of melanosomes in the feathers, rendering it impossible to isolate a region bearing only melanosomes for analysis. ToF-SIMS spectra of the melanin extracts (Supplementary Fig. 10) are also dominated by fragment ions for proteins and lipids, with additional peaks for phosphate (Supplementary Figs. 11, 12). In these samples, the protein fragments likely derive from the melanosome membrane and/or protein residues that are adhering to the melanosome surfaces. In turn, these protein residues probably derive from the enzymatic extraction process. The phosphate peaks derive from the phosphate buffer used during the extractions. Collectively, these data suggest that ToF-SIMS is not the most appropriate technique to use for chemical analysis of these biological tissues: the spectral data are effectively swamped by other fragment ions. It is important to note that ToF-SIMS has been applied successfully to analysis of pure synthetic standards of selected eumelanin monomers and their degradation products generated during maturation experiments^25^; the resulting ToF-SIMS data are entirely consistent with AHPO-HPLC data on the samples, confirming the accuracy and validity of AHPO-HPLC for investigation of chemical alteration of melanins.

### Melanin chemistry in fossils

**Fig. 4:**
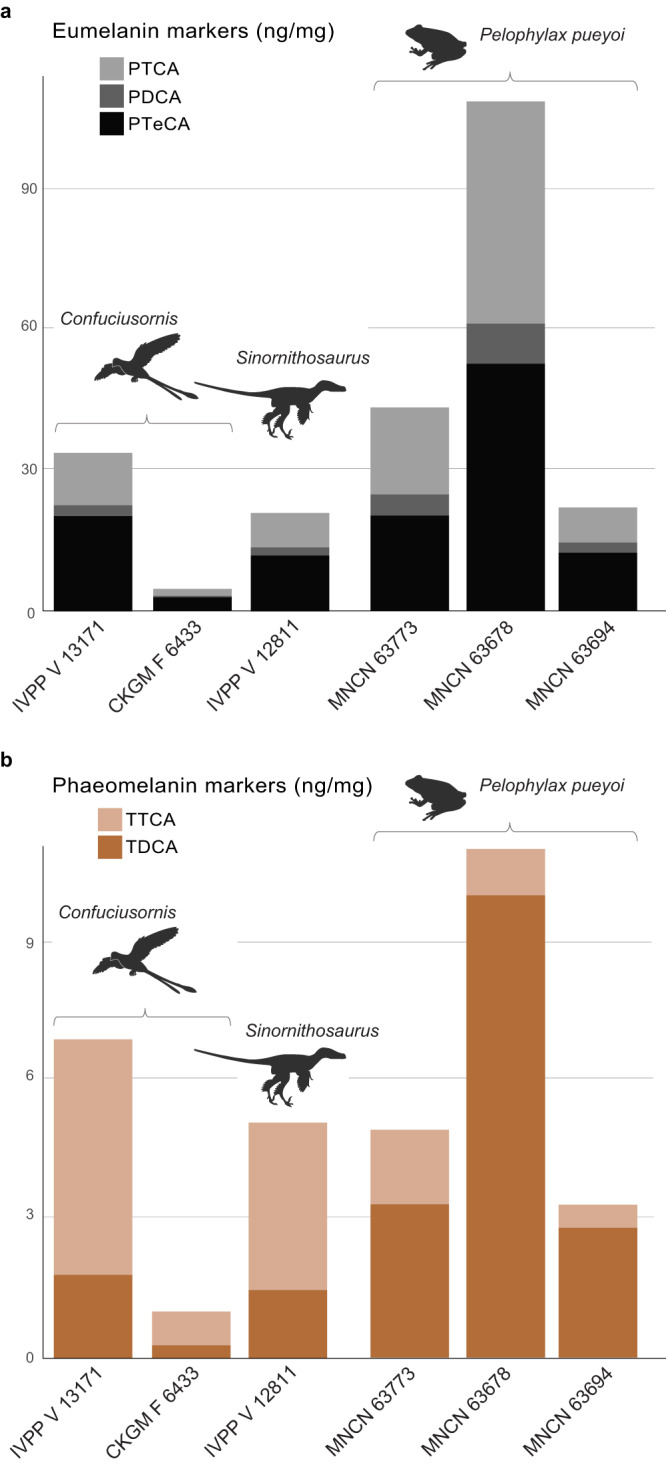
HCl-AHPO-HPLC data for fossil melanins. Eumelanin (**a**) and phaeomelanin (**b**) marker concentrations in samples of melanosomes. Fossil feathers are from the early bird *Confuciusornis* and the feathered dinosaur *Sinornithosaurus* from the Jehol biota. Fossil frog samples are of internal melanosomes from *Pelophylax pueyoi* from the Libros biota. PTCA pyrrole-2,3,5-tricarboxylic acid, PDCA pyrrole-2,3-dicarboxylic acid, PTeCA pyrrole-2,3,4,5-tetracarboxylic acid, BZ benzothiazole, TTCA thiazole-2,4,5-tricarboxylic acid, TDCA thiazole-4,5-dicarboxylic acid. Source data are provided as a Source Data file. *Sinornithosaurus* and *Confuciusornis* silhouettes by Conty (modified by T. Slater). Used under an Attribution 3.0 Unported (CC by 3.0) license. https://creativecommons.org/licenses/by/3.0/.

HCl-AHPO-HPLC analysis of fossil melanosomes (Fig. 4) reveals that melanin markers are most abundant in the internal melanosomes from the Miocene frogs. Eumelanin marker concentrations are typically 21.8–60.8 ng/mg but are particularly high in CKGM F 6434 (354.1 ng/mg); high concentrations have been reported in other fossils^34^. Marker concentrations are much lower in the sedimentary matrix (Table 3), indicating that the markers in the fossil soft tissues are autochthonous and do not derive from the sediment. In the other frog samples, PTCA (7–26 ng/mg, mean 18 ng/mg) and PTeCA (12–31 ng/mg, mean 21 ng/mg) are similar and PDCA is a minor component (2–5 ng/mg, mean 4 ng/mg). Concentrations of eumelanin markers are lower in the fossil feathers relative to the frogs and show consistently higher concentrations of PTeCA relative to PTCA and especially PDCA (PTeCA: 2.6–12.8 ng/mg, mean 6.7 ng/mg; PTCA: 1.5–7.3 ng/mg, mean 2 ng/mg; PDCA: 4 ng/mg). In general, the PTeCA: PTCA ratio is higher for markers from the fossil feathers (mean 1.66) than frogs (mean 1.21), which suggests a greater degree of polymerisation in the fossil feathers^34^.

**Table 3:** HCl-AHPO-HPLC data for individual melanin marker concentrations (ng/mg) in fossil soft tissues and host sediments

	Eumelanin markers				Phaeomelanin markers		
Fossil	PTCA	PDCA	PTeCA	PTeCA: PTCA	TTCA	TDCA	TTCA: TDCA
*Sinornithosaurus* IVPP V 12811	3.71 ± 1.08	0.79 ± 0.04	5.78 ± 1.88	1.54 ± 0.06	3.13 ± 0	1.13 ± 0	2.77 ± 0
Sediment IVPP V 12811	1.05	0.52	2.26	2.15	<0.3	<0.2	1.5
*Confuciusornis* IVPP V 13171	5.72 ± 1.54	1.02 ± 0.15	10.02 ± 2.78	1.75 ± 0.01	4.59 ± 0	1.47 ± 0	3.12 ± 0
*Confuciusornis* CKGM F 6433	1.51	0.29	2.59	1.72	0.73	0.25	2.92
Sediment CKGM F 6433	0.70	0.16	1.14	1.63	0.11	0.2	0.55
*Pelophylax pueyoi* (adult) MNCN 63773	18.6	4.61	20.0	1.08	1.62	3.29	0.49
*Pelophylax pueyoi* (adult) MNCN 63678	23.75 ± 3.05	4.43 ± 0.51	26.15 ± 6.58	1.09 ± 0.13	–	4.99 ± 1.29	–
*Pelophylax pueyoi* (adult) MNCN 63694	7.51	2.31	12.0	1.6	<0.5	2.79	0.18
*Pelophylax pueyoi* (tadpole) CKGM F 6434	155	30.1	169	1.09	45.4	42.1	1.08
Sediment CKGM F 6434	4.84	5.42	13.4	2.77	11.4	5.29	2.16

*PTCA* pyrrole-2,3,5-tricarboxylic acid, *PDCA* pyrrole-2,3-dicarboxylic acid, *PTeCA* pyrrole-2,3,4,5-tetracarboxylic acid, *TTCA* thiazole-2,4,5-tricarboxylic acid, *TDCA* thiazole-4,5-dicarboxylic acid. Source data are provided as a Source Data file.

The phaeomelanin markers TTCA and TDCA are detected in low quantities in both fossil feathers and frogs (Fig. 4). The markers are below detection limits for two feather samples; in the remaining three feather samples, TTCA is consistently more abundant than TDCA (TDCA: 0.25–1.5 ng/mg, mean 0.95 ng/mg; TTCA: 0.7–4.6 ng/mg, mean 2.82 ng/mg). These marker values are substantially higher than the values in the host sediment (Table 3). This strongly suggests minimal potential contributions from exogenous sources but values are not sufficiently high to exclude potential contributions from endogenous, non-melanin, feather components. Indeed, the higher concentrations of TTCA relative to TDCA are more consistent with a proteinaceous source rather than feather melanin. In contrast, in the fossil frog samples, TDCA is consistently more abundant than TTCA (TDCA: 2.8–5.9 ng/mg, mean 4 ng/mg; TTCA: below detection limit–1.6 ng/mg) except in CKGM F 6434, where TTCA ≈ TDCA.

### Predictive model for fossil melanin chemistry

**Fig. 5:**
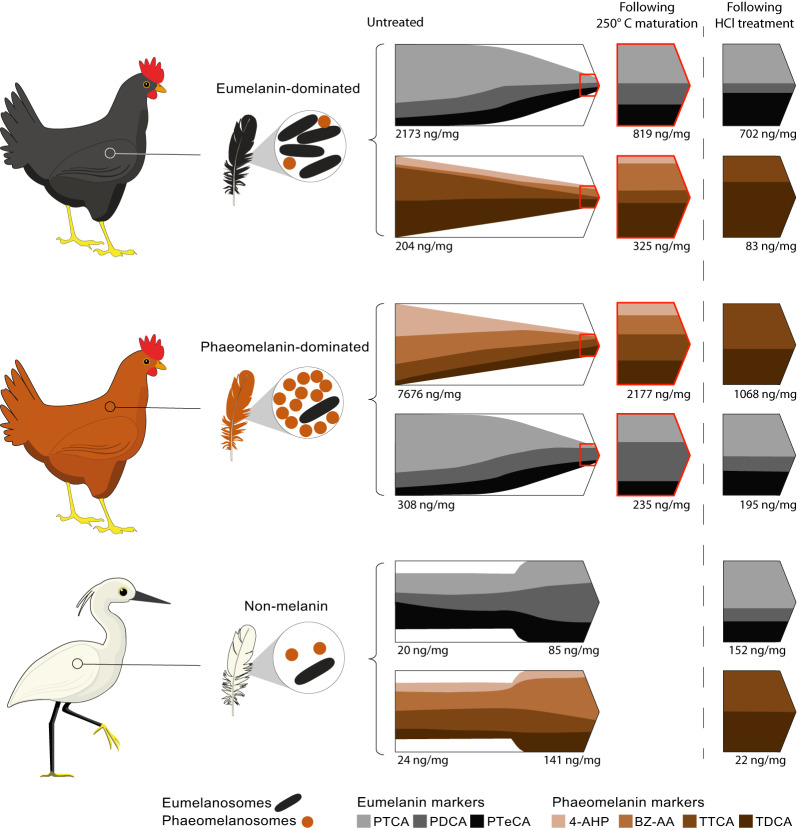
Authentic chemical signals for fossil melanins. Schematic showing chemical degradation of eumelanin and phaeomelanin during fossilisation based on AHPO-HPLC analysis of experimentally matured black and rufous (*Gallus gallus*) and white (*Egretta garzetta*) feathers. Dashed line separates data with, and without, HCl treatment. The long horizontal arrows illustrate the relative concentrations of melanin markers before treatment (left-hand margin of arrow) and after maturation at 250 °C (right-hand margin (point) of arrow) without HCl treatment. The short horizontal arrows (on the right) depict the relative concentrations of markers following HCl treatment and maturation at 250 °C. Concentration values (in ng/mg) indicate absolute concentrations of melanin markers. PTCA pyrrole-2,3,5-tricarboxylic acid, PDCA pyrrole-2,3-dicarboxylic acid, PTeCA pyrrole-2,3,4,5-tetracarboxylic acid, 4-AHP 4-amino-3-hydroxyphenylalanine, BZ-AA benzothiazole amino acid, TTCA thiazole-2,4,5-tricarboxylic acid, TDCA thiazole-4,5-dicarboxylic acid. *Egretta garzetta* by Punnawich Limparungpatanakij (modified by T. Slater). Used under a Royalty Free license. https://dreamstime.com.

Our HPLC data form the basis of a predictive model for authentic signals for eumelanin and phaeomelanin in fossil tissues (Fig. 5 and Table 4). We first present the model for strongly matured samples analysed with AHPO-HPLC, and then show how the model predictions are modified by acid treatment. In our model, the key markers for eumelanin are PTCA and PTeCA (as the relationship between the two is an indicator of the extent of maturation^37^); those for phaeomelanin are TTCA and TDCA (as BZ-AA and 4-AHP have yet to be analysed using HI hydrolysis-HPLC).

**Table 4:** Predictions of authentic signals for fossil melanins in eumelanin- and phaeomelanin-dominated systems

		No HCl treatment		HCl treatment
Feather type	Type of markers	Moderate maturation	Strong maturation	Strong maturation
Eumelanin-dominated	Eumelanin	PTCA ≫ PTeCA > PDCA	PTCA > PDCA ≥ PTeCA	PTeCA ≈ PTCA ≫ PDCA
	Phaeomelanin	TDCA > TTCA	TDCA > TTCA	TDCA ≫ TTCA
Phaeomelanin-dominated	Eumelanin	PTCA > PDCA > PTeCA	PDCA > PTCA > PTeCA	PTCA > PTeCA > PDCA
	Phaeomelanin	TTCA ≫ TDCA	TTCA ≥ TDCA	TDCA > TTCA
Non-melanin	Eumelanin	PTCA ≈ PTeCA > PDCA	PDCA > PTCA > PTeCA	PTCA > PTeCA ≫ PDCA
	Phaeomelanin	TTCA > TDCA	TDCA ≈ TTCA	TTCA ≈ TDCA

Predictions are based on AHPO-HPLC and HCl-AHPO-HPLC data from untreated and experimentally matured black and rufous (*Gallus gallus*) and white (*Egretta garzetta*) feathers. These feathers represent eumelanin-dominated and phaeomelanin-dominated systems, respectively. Data for non-melanin systems indicate signals for diagenetic artefacts produced by thermal maturation of melanin-poor, protein-rich systems. Predictions for moderate maturation are derived from the data from the 200 °C experiment; predictions for strong maturation are derived from trends extrapolated from the 250 °C experiment.

The chemistry of fossil melanins can be predicted by examining changes in the relative proportions of the different melanin markers during experimental maturation (Figs. 2, 3 and Supplementary Figs. 2, 3). Where samples have not been acid treated, our experimental data indicate that an authentic signal for strongly matured eumelanin should show PTCA > PDCA ≥ PTeCA. This is supported by an accessory phaeomelanin signal dominated by BZ-AA and TDCA, with minor TTCA and especially 4-AHP (i.e., BZ-AA > TDCA > > TTCA > 4-AHP and the key signal TDCA > TTCA).

Authentic signals for strongly matured phaeomelanin should exhibit abundant TTCA and TDCA, with BZ-AA and 4-AHP as minor components, i.e., TTCA ≥ TDCA > BZ-AA > 4-AHP. This is supported by an accessory eumelanin signal dominated by PDCA with minor PTCA and PTeCA, i.e., PDCA > PTCA > PTeCA.

Data from matured white feathers show that most melanin markers increase in concentration when matured at 250 °C, with eumelanin markers dominant. The relative proportions of PDCA and, to a lesser extent, PTCA also increase. These changes are not consistent with thermal degradation of melanin, but rather suggest minor generation of melanin monomers from non-melanin compounds. An artificial melanin signal in fossils will thus be dominated by eumelanin markers, with PDCA > PTCA > PTeCA. Accessory phaeomelanin markers will be dominated by BZ-AA, with minor amounts of all other markers, i.e., yielding BZ-AA ≫ TTCA ≈ TDCA > 4-AHP (i.e., TDCA ≈ TTCA).

The predictive model requires modifications for HCl-treated samples (Fig. 3 and Supplementary Fig. 3). In eumelanin-dominated systems, strongly matured HCl-treated samples generate a signal dominated by eumelanin markers, especially PTeCA and PTCA (in similar proportions) with minor PDCA. An authentic signal for strongly matured eumelanin after HCl treatment of samples should therefore show PTeCA ≈ PTCA ≫ PDCA, with accessory TDCA ≫ TTCA.

HCl-treated and strongly matured samples from phaeomelanin-dominated systems should be dominated by phaeomelanin markers and should contain more TTCA than TDCA, i.e., TTCA > TDCA, with an accessory eumelanin signal showing PTCA > PTeCA > PDCA.

An artificial melanin signal will be dominated by eumelanin markers, with PTCA > PTeCA ≫ PDCA; phaeomelanin markers will be minor components (with TTCA ≈ TDCA).

### Testing the model: fossil melanosomes and melanin chemistry

The fossil frog melanosomes analysed are ovoid (0.75–1.2 μm long) to spherical (0.40–0.55 μm long)^50^. Preserved melanosome morphology, however, is unlikely to inform on original melanin chemistry in these specimens: melanosomes in extant amphibians lack differentiation into strongly elongate and spherical forms, which is likely a feature ancestral to the group^4,20^. The HCl-treated fossil frog melanosomes are dominated by eumelanin markers, especially PTeCA and PTCA, with PTeCA ≈ PTCA; except in CKGM F 6434 (where TDCA ≈ TTCA), phaeomelanin markers are consistently dominated by TDCA (Fig. 4; Table 3). The HCl-AHPO-HPLC signal in the fossil data is therefore not consistent with artificially generated eumelanin (where the model predicts PTCA > PTeCA ≫ PDCA) nor with an original composition dominated by phaeomelanin (where the model predicts dominant phaeomelanin markers and accessory PTCA > PTeCA > PDCA). Instead, the eumelanin marker data are consistent with an original eumelanic composition (where PTeCA ≈ PTCA is predicted). This is supported by the eumelanin-dominated composition of melanosomes in extant amphibians^20^. The dominant phaeomelanin signal in the fossil frogs (TTCA < TDCA) is not consistent with artificial generation from non-melanin components, for which the model predicts TTCA ≈ TDCA in HCl-treated fossil samples. Instead, the fossil phaeomelanin signal is consistent with an original accessory phaeomelanin component, for which the model predicts TTCA < TDCA. This is supported by the presence of minor quantities of phaeomelanin in melanosomes of extant amphibians^20^ and by the presence of elevated concentrations of Zn in melanosomes from the Libros frogs^21^, which is indicative of phaeomelanin^12^.

The fossil feather melanosomes are dominated by eumelanin markers, with PTeCA > PTCA ≫ PDCA, and an accessory phaeomelanin signal with TTCA > TDCA. Melanin markers are also present in the host sediment, albeit at much lower levels than in the soft tissues of the corresponding specimen (Table 3), indicating that the markers in the fossil soft tissues do not derive from the sediment. The bulk AHPO signal is not consistent with a phaeomelanin-dominated system; further, the eumelanin signal is not consistent with artificial generation of melanin (where the model predicts PTCA > PTeCA > PDCA in HCl-treated samples). Instead, the fossil feather signal is most consistent with an original eumelanin-rich system. The higher PTeCA: PTCA ratio in the fossil feathers relative to the model (which predicts PTeCA ≈ PTCA, with a slight dominance of PTeCA over PTCA) probably reflects more extensive cross-linking of DHI units during fossilisation over long geological timescales. This suggests that DHI is more robust than DHICA during thermochemolysis^34,37,38^.

An original eumelanin component in the fossil feathers is supported by the presence of rod-shaped melanosomes—presumably eumelanosomes—at least locally in *Confuciusornis*^9,51^. Given reports of abundant melanosomes with ovoid to spheroid geometries (Supplementary Fig. 13a)—presumably phaeomelanosomes^9,51^—in *Confuciusornis*, it is somewhat surprising that phaeomelanin markers are very low in concentration (although the relative proportions of presumed eumelanosomes to phaeomelanosomes in *Confuciusornis* has not been estimated). This is consistent with our experimental data that demonstrate enhanced degradation of phaeomelanin relative to eumelanin during thermal maturation and is supported by the diagenetic history of the host sediment: fossils from the Jehol biota experienced relatively high burial temperatures^32^. Other potential contributing factors include oxidation during diagenesis^52^, which may preferentially degrade phaeomelanin^32^; our experimental data presented here also suggest a further bias against recovery of phaeomelanin markers from HCl-treated samples. Given these biases, it is plausible that the *Confuciusornis* feathers analysed originally had a higher proportion of phaeomelanin than the eumelanin-dominated feathers matured in our experiments. This could also reconcile the TTCA > TDCA signal in the fossil feathers with our model, which is consistent with a phaeomelanin-dominated composition (it is also consistent with artificial marker generation, but the dominance of PTeCA over PTCA in the fossil feathers renders this unlikely).

### Broader implications

Our study provides empirical and comparative models for the degradation of eumelanin and phaeomelanin during thermal maturation and, in doing so, identifies authentic molecular signals for these forms of melanin in fossils. Analysis of fossil melanosomes, combined with a holistic consideration of preserved melanosome morphology and the chemistry of melanosomes in extant analogues, confirms the validity of our model. Our experiments demonstrate that phaeomelanin monomers can survive strong maturation and can be detected in thermally matured tissues using (HCl-)AHPO-HPLC. Critically, our study provides strong empirical support for the survival of phaeomelanin in Miocene fossils (for which chemical data are fully consistent with our model). Our data also suggest the preservation of phaeomelanin in Mesozoic fossils that have survived extensive thermal degradation and/or oxidation.

Our experiments use a simple model with near-pure natural end members (in terms of melanin chemistry) and a reductionist approach to maturation experiments, to facilitate the interpretation of results. Natural biological systems and diagenesis are clearly more complex. For instance, the absolute, and relative, abundance of melanin markers in experimentally matured and fossil feathers may vary due to differences in the original concentrations of melanin (and potentially other pigments, e.g., porphyrins) among feathers of a single species or among species. Further work is required to characterise the confounding effects of diagenesis over long intervals and/or high temperatures, oxidation and HCl treatment on feathers with different proportions of melanin markers.

Differences in the response of eumelanin and phaeomelanin markers to thermal maturation in eumelanin- and phaeomelanin-rich feathers, respectively, highlight the importance of the local chemical environment on the degradation of melanin. Interpretations of melanin chemistry in fossils should therefore apply a holistic approach to data interpretation that combines molecular data with data on the morphology of associated fossil melanosomes and on melanosome morphology and melanin chemistry in extant relatives.

The increase in 4-AHP and BZ-AA in black feathers, and of all melanin markers in white feathers during strong maturation, probably reflects artificial formation due to degradation of proteins and/or other non-melanin components. Concentrations of these artificial melanin markers are especially high where samples are treated with HCl. The formation of markers from non-melanin components is unlikely to make a major contribution to data from fossil eumelanin as concentrations of artificially generated eumelanin markers are predicted to be much lower than for originally eumelanin-rich fossils, even after HCl treatment. Particular caution is required, however, when interpreting data on phaeomelanin markers in fossils, as our data suggest that such markers can be readily generated during HCl treatment of melanin-poor tissue samples. Interpretations of authentic fossil phaeomelanin are, however, supported by preservation of melanosomes, especially those with spheroidal shapes in feathers, and by consideration of melanin chemistry in relevant tissues in extant analogues. Future studies on fossil melanins should clearly incorporate an understanding of the potential impact of diagenetic artefacts on molecular data.

Emerging data on taxonomic and tissue-specific trends in the distribution of melanin in vertebrates suggest important shifts in the functions of melanin in immunity, homeostasis and visual communication over geological time^4^. It is therefore critical that studies of the macroevolutionary history of melanin and its functions are not undermined by controversy regarding the validity of the fossil evidence^23,53^. Our results provide a framework for the interpretation of fossil melanosome chemistry, thus constraining future hypotheses on melanin evolution through deep time.

## Methods

### Maturation experiments

Replicate black (*n* = 5) and rufous (*n* = 5) contour feathers from the domestic chicken, *Gallus gallus domesticus*, and white (*n* = 4) primary feathers from a wild specimen of the little egret, *Egretta garzetta*, were stored at -80 °C prior to experimental treatment to prevent decay. Feathers from *Gallus gallus* were naturally shed and then donated by private individuals; feathers from *Egretta garzetta* were collected from a deceased egret found in an urban area of Cork, Ireland. Feathers were selected based on their high content of eumelanin or phaeomelanin (black and rufous feathers, respectively), or near-lack of pigmentation (white feathers)^6^. Previous studies have shown that white feathers may contain very minor amounts of non-melanin pigments^54^. Although not reported in egrets, we cannot completely exclude the possibility that the white egret feathers used in this study contain minor amounts of non-melanin pigments. A sample (ca. 45 × 25 mm) of the feather vane comprising barbs with interlocking barbules was sampled from each feather, wrapped in Al foil and thermally matured in a standard laboratory convection oven for 1 h at 100 °C, 200 °C or 250 °C (each +/- 1 °C; Supplementary Table 1).

### Experimental justification

For any taphonomic experiment, the optimum conditions are those that generate morphological and/or chemical phenomena that are similar to features of interest in fossils^52^. In this study, the fossils of interest are organically preserved fossil feathers that retain gross feather structure (e.g., rachis and barbs) and melanosomes. The purpose of our experiments is to investigate the impact of thermal maturation on the chemistry of feather melanin, and then to apply the experimental results to the interpretation of melanin chemistry in fossil feathers. The most appropriate conditions for our experiments are therefore those that ensure the survival of melanosomes and the surrounding proteinaceous tissue (or at least residues thereof) at the end of the experiment^32,52^ yet involve sufficiently high temperatures and/or pressures to ensure geochemical alteration of melanin. In other words, the experiments should achieve a taphonomic sweet spot between too much, and too little, degradation.

#### Experimental maturation and feather structure

Previous maturation experiments on feathers have used various combinations of pressure and temperature, with feathers enclosed in foil or Au capsules. Substantial deterioration of feather macrostructure (including a reduction in volume, merging of barbs, and contortion and loss of barbules) occurs after 1 h in foil at 200 °C, 200 bar (following a 1 h-long ramp-up of temperature to 200 °C^55^) and 1 h in foil at 200 °C, 250 bar^32^. Fluidization of feather tissue occurs after 24 h in Au capsules at 250 °C, 250 bar^56^. All of these conditions are clearly too extreme for the purposes of our experiments. In contrast, experiments using lower temperatures have reported preservation of feather macrostructure with little or no appreciable damage, e.g., with feather samples in Au capsules for 24 h at 100 °C, 250 bar^56^ and, in separate experiments, in aluminium foil for 1 hr^55^. Use of low pressures, even when coupled with higher temperatures, promotes retention of intact feather structures, as in previous experiments for 1 h at 200 °C, 135 bar^52^. Collectively these data suggest that for our study (with temperature as the key variable), retention of feather macrostructure will be best achieved in experiments at relatively low pressures, i.e., at 135 bar or less.

#### Maturation experiments and melanin chemistry

Previous experiments have not systematically analysed melanin monomer chemistry (but see ref. ^52^). Conditions promoting geochemical alteration of melanosomes can, however, be inferred from published data. Previous experiments at 100 °C, 250 bar for 24 h did not result in major differences between the chemistry of matured and untreated feathers, as evidenced by gas chromatography-mass spectrometry analyses^56^. These experimental temperatures are clearly too low to induce substantial chemical alteration of melanosomes, especially since elevated pressures enhance degradation of tissue ultrastructures^57^. We tested this using a set of pilot experiments on rufous feathers at 100 °C for 1 h (Supplementary Text and Supplementary Figs. 14, 15 and Table 2). As predicted, after maturation, the TTCA: TDCA ratio is unchanged in HCl-treated samples (0.71 for both untreated and matured samples) and very similar in samples lacking acid treatment (0.93 (untreated) and 0.88 (matured)); the 4-AHP: BZ-AA ratio is similar in untreated (0.5) and matured (0.6) samples lacking acid treatment.

Experiments at 200 °C, 250 bar and 250 °C, 250 bar for 24 h induced shrinkage of melanosomes^32^ and altered melanosome chemistry^27^. Such elevated temperatures are therefore optimal for our experiments, albeit not in conjunction with elevated pressures, as this combination induces substantial feather degradation (see above). Our experiments therefore used relatively elevated temperatures of 200 °C and 250 °C and atmospheric pressure. Although our experiments were conducted in air and can thus be classed as broadly oxidising, they did not incorporate reaction with a strong oxidant as our previous experiments have shown that this destroys melanosomes^52^.

Chemical analysis of thermally matured melanosomes can inform on the geochemistry of preserved melanosomes associated with eumelanin- and phaeomelanin-rich fossil feathers. White feathers were used to test whether markers generated by AHPO and HI hydrolysis are unique to melanin. Experiments were performed under oxic conditions to enhance thermal degradation (relative to anoxic conditions^57^). No additional experimental media were used as the focus of the experiments is the analysis of feather residues resulting from autochthonous in-situ chemical changes, not changes induced by extrinsic fluids.

### Fossil material

This study used several specimens from the Jehol (Early Cretaceous, NE China) and Libros (Late Miocene, NE Spain) biotas. Fossils analysed from the Jehol Biota are two specimens (CKGM F 6433 and IVPP V 13171) of *Confuciusornis* and one specimen of *Sinornithosaurus millenii* (IVPP V 12811). Fossils from Libros are four specimens of *Pelophylax pueyoi* (CKGM F 6434, MNCN 63678, MNCN 63694 and MNCN 63773). Permission for destructive sampling was granted by museum curators. Samples were taken of feathers from dinosaur specimens and of internal melanosomes from frogs; fossil soft tissues and host sediments were sampled with sterile scalpels and forceps. The fossil frog soft tissues are appropriate for comparison with the fossil feathers because SEM images of the former comprise exclusively melanosomes; there is no evidence for any other tissue components (Supplementary Fig. 13). Further, chemical data from py-GC-MS and ToF-SIMS analyses^29^ show evidence for melanin and abundant aliphatic and organosulfur compounds (consistent with existing models for in situ polymerisation of organic fossils and kerogen^29^), but no evidence for degradation products of proteins or other tissue components. The fossil frog soft tissues are therefore effectively an excellent natural standard for fossil melanin and suitable for comparison with other fossil melanosomes. Institutional abbreviations: CKGM, Cork Geological Museum; IVPP, Institute of Vertebrate Paleontology and Paleoanthropology, Beijing, China; MNCN, Museu Nacional de Ciencias Naturales, Madrid, Spain.

### SEM

Samples of preserved soft tissue were dissected from fossil specimens using sterile tools, mounted on Al stubs with carbon tape, sputter coated with C or Au and analysed using a Hitachi S-3500N scanning electron microscope at accelerating voltages of 15–25 kV.

### HPLC analyses

Feather samples (each 3–5 mg) were homogenised in distilled water with a Ten-Broeck glass homogeniser at a concentration of 5 mg/mL. Separate aliquots (100 µL) of this homogenate were subjected to AHPO, HI hydrolysis or Soluene-350 solubilisation (the last on untreated black and rufous feathers only: black and rufous, each *n* = 3).

AHPO tests for the presence of the eumelanin markers PTCA, PDCA and PTeCA and the phaeomelanin markers TTCA and TDCA^36,37^. Aliquots of homogenised feather samples were subjected to AHPO by adding 375 µl 1 M K_2_CO_3_ and 25 µl 30% H_2_O_2_ and placed in a test-tube mixer at 25 °C + /- 1 °C for 20 h^36,37^; the reaction was terminated by adding 150 µl 6 M H_3_PO_4_^58^. Fossil and host sediment samples (1–8 mg each) were ground to a powder and subjected to HCl-AHPO by adding 0.5 ml 6 M HCl and heating at 110 °C for 16 h^46^. HCl-AHPO was developed in order to demineralise samples and purify analytical results by removing interfering materials such as proteins^46^.

HI hydrolysis tests for the phaeomelanin markers 3-AHP, 4-AHP^41^ and BZ-AA^42^. 3-AHP is a minor component of phaeomelanin degradation products^41^ and can be derived from proteins (i.e., it is not melanin-specific)^59^; 3-AHP data are thus not a major focus here (for details see Supplementary Text and Supplementary Fig. 16). HI hydrolysis was performed by heating the aliquots of homogenised feather samples with 30 µl 30% H_3_PO_2_ and 500 µl 57% HI at 130 °C for 20 h. The mixtures were then cooled and dried using a vacuum pump equipped with an ice-cooled vacuum trap and two filter flasks containing NaOH pellets; residues were dissolved in 200 µl 0.1 M HCl^41^.

Products from AHPO, HCl-AHPO and HI hydrolysis were analysed using an HPLC system comprising a JASCO 880-PU pump (JASCO Co., Tokyo, Japan), a C18 column (Capcell Pak MG; 4.6 × 250 mm; 5 µm particle size, Osaka Soda, Osaka, Japan) and a JASCO UV detector (JASCO Co., Tokyo, Japan) at 272 nm for PTCA, PDCA, TTCA and TDCA and at 269 nm for PTeCA, PTCA and PDCA^58^. BZ-AA and 4-AHP cannot be analysed following acid treatment. A different HPLC column was used to analyse feathers matured at 100 °C than other feather samples (i.e., untreated feathers and feathers matured at 200 °C and 250 °C) due to regular replacement of the column as per standard protocols. Identification of melanin markers was confirmed by co-injecting a melanin standard in the HPLC and comparing retention times, and ratios of peak area/height, of the standard with the experimental data. The only melanin marker that is difficult to identify with confidence is PDCA as it shows similar retention times to certain degradation products of proteins. The presence of PDCA was confirmed by repeating the analysis at a higher HPLC column temperature, which separates the peak for PDCA from any interfering peaks derived from proteins. All other melanin markers, especially PTeCA and TTCA, are readily identified as their retention times are distinct to those of other AHPO products.

Soluene-350 solubilization was used to determine spectrophotometric A500 and A650 values, which were subtracted from background values of 0.021/mg and 0.001/mg, respectively^59^. To each aliquot of homogenised feather sample 900 µl of Soluene-350 was added and the test tubes were mixed. Test tubes were then placed in a boiling water bath for 30 min, cooled, mixed and placed in a boiling water bath for 15 min. The solutions were transferred to Eppendorf tubes and centrifuged at 10,000 rpm for 10 min. Supernatants were analysed using a spectrophotometer at 500 nm and 650 nm.

Values cited herein for percentage change in monomer concentrations (ng/mg) for experimentally matured feathers of a particular colour are relative to mean concentrations of that monomer in untreated feathers of the same colour.

### ToF-SIMS

Zebra finch (*Taeniopygia guttata*) were sourced from animal suppliers and euthanasia was either approved by the Health Products Regulatory Authority of Ireland via authorization AE19130-IO87 for black and rufous feathers or performed in accordance with Swedish regulations for rufous feathers. Samples of zebra finch melanin were obtained using the enzymatic protocols in refs. ^20^ (see Supplementary Fig. 10) and ^60^ (see Supplementary Fig. 4) on untreated black (*n* = 1) and rufous (*n* = 2) contour feathers. In brief, feather samples subjected to the protocol in ref. ^20^ were treated with solutions of 1,4-DTT, proteinase-K and papain at 37.5 °C and 200 rpm for nine cycles, each nine days; feather samples processed according to the protocol in ref. ^60^ were incubated with a mixture of 1,4-DTT, proteinase-K and papain and stirred at 37.5 °C for 4 days. Melanin from the cuttlefish (*Sepia officinalis*) was purchased from Sigma-Aldrich. Synthetic eumelanin was purchased from Fisher Scientific, washed with ultrapure water and ethanol five times each and dried before use. Synthetic phaeomelanin was produced using the protocol in ref. ^61^.

For ToF-SIMS analysis, samples of melanin extracts and standards were mounted on ToF-SIMS sample holders. ToF-SIMS analyses were conducted in the static SIMS mode on a TOFSIMS IV instrument (IONTOF GmbH) using 25 keV Bi^3+^ primary ions and low energy electron flooding for charge compensation. Positive and negative ion data were acquired with instrument optimization for high mass-resolution (m/Δm ~5000, spatial resolution ~3–4 μm). The pulsed primary ion current was set at 0.10 pA.

The primary results reported here derive from AHPO-HPLC analysis, which is the standard for analysis of melanins in modern tissue samples because it detects and discriminates peaks for melanin monomers, i.e., it is highly specific. Further, HPLC is readily accessible and relatively inexpensive. AHPO-HPLC is, however, destructive, so it is not suitable for certain fossils, especially where soft tissues are limited in extent and/or for small specimens.

We include data from ToF-SIMS for comparison as this approach has previously been applied to melanosome-rich soft tissues from other fossils. ToF-SIMS is less diagnostic than AHPO-HPLC as it is based on statistical analysis of assemblages of small fragment ions that individually have little diagnostic value. ToF-SIMS is, however, particularly useful for analysis of fossil melanosomes, in which melanin has been concentrated during diagenesis due to preferential degradation of proteins and lipids in melanosomes^26^; the technique is also nondestructive. ToF-SIMS data are less readily interpreted for biological samples where the compound of interest is highly dispersed and/or not exposed at the surface (the technique is highly surface sensitive).

### Development of the predictive model

The predictive model is based on data for (a) mean concentrations of individual melanin markers per treatment; (b) standard deviation data for individual markers per treatment (with particular attention to overlap of the range of the data for different treatments); (c) trends in the data for relative concentrations of individual markers (i.e., when expressed as a percentage of the total markers recovered for eumelanin and phaeomelanin, respectively, in a sample). To illustrate, consider data from strongly matured eumelanin-dominated systems analysed using HCl pre-treatment. PTCA concentrations are predicted to exceed those for PDCA based on the higher quantities of PTCA remaining following treatment relative to PDCA. With progressive maturation beyond the extent modelled in our experiments, PTeCA may ultimately dominate the melanin signal in fossils given the increase in the PTeCA: PTCA ratio during maturation which is observed in our AHPO-HPLC data and has been reported previously^37^.

### Reporting summary

Further information on research design is available in the Nature Portfolio Reporting Summary linked to this article.

### Supplementary information

Supplementary Information

Reporting Summary

### Source data

Source Data

## Data Availability

The HPLC data generated in this study are provided in the Source Data file. Source data are provided with this paper.
