# Supplementary material for: Taphonomic experiments reveal authentic molecular signals for fossil melanins and verify preservation of phaeomelanin in fossils
Source: Nat Commun. 2023 Oct 6;14:5651. doi: 10.1038/s41467-023-40570-w (PMC10558522; doi:10.1038/s41467-023-40570-w)
Supplement: Supplementary file 1 — Supplementary Information [file 41467_2023_40570_MOESM1_ESM.pdf]

Supplementary information for

Taphonomic experiments reveal authentic molecular signals for fossil melanins and verify preservation of phaeomelanin in fossils

Tiffany S. Slater[1,2*], Shosuke Ito[3], Kazumasa Wakamatsu[3], Fucheng Zhang[4], Peter Sjövall[5], Martin Jarenmark[6], Johan Lindgren[6] and Maria E. McNamara[1,2*]

[1]School of Biological, Earth and Environmental Sciences, University College Cork, Cork, Ireland. [2]Environmental Research Institute, University College Cork, Cork, Ireland. [3]Institute for Melanin Chemistry, Fujita Health University, Toyoake, Aichi, Japan. [4]Institute of Geology and Paleontology, Linyi University, Linyi City, Shandong, China. [5]RISE Research Institutes of Sweden, Materials and Production, 501 15 Borås, Sweden. [6]Department of Geology, Lund University, 223 62 Lund, Sweden. email: tiffany.slater@ucc.ie; maria.mcnamara@ucc.ie

Supplementary Text
Supplementary Figures 1–16
Supplementary Tables 1 and 2
Supplementary references

Supplementary Text

3-AHP. HI-HPLC data show small quantities of 3-AHP in untreated black (13 ng/mg) and white (4 ng/mg) feathers and large quantities in rufous feathers (1341 ng/mg; Supplementary Fig. 16). 3-AHP increases with progressive maturation in black (200°C: 22 ng/mg; 250°C: 39 ng/mg) and white (200°C: 11 ng/mg; 250°C: 70 ng/mg) feathers but decreases in rufous feathers (200°C: 587 ng/mg; 250°C: 152 ng/mg). The progressive increase in black and white feathers likely reflects minor generation of this moiety from non-melanin feather components.

Melanin chemistry of rufous feathers matured at 100°C. The purpose of this supplementary experiment was to determine whether a temperature of 100°C is sufficiently high to result in substantial degradation of phaeomelanin following thermal maturation. Results are thus reported here for phaeomelanin markers (data for accessory eumelanin markers are provided in Supplementary Figs. 14, 15 and Table 2). Rufous feathers matured at 100°C (n = 3) show relatively minor changes in concentrations of TTCA (1395 ng/mg, +35%) and TDCA (104 ng/mg, -25%) compared to untreated feathers. These data are not consistent with thermally-induced decarboxylation of TTCA to TDCA and may reflect minor variation in feather chemistry (especially BZ) among samples and/or feathers. HI-HPLC data show a minor decrease in both 4-AHP (3320 ng/mg, -15%) and BZ-AA (2121 ng/mg, -18%) relative to untreated feathers. These data indicate only minor degradation of BT (which results in lower 4-AHP values) and no change in BZ (degradation of BZ results in lower TDCA values) relative to untreated feathers. HCl-AHPO shows an increase in TTCA (608 ng/mg, +71%) and TDCA (244 ng/mg, +70%). The higher HCl-TTCA values than in untreated feathers may reflect slightly higher intrinsic BZ values in the samples matured at 100°C. The increase in HCl-TDCA, while marked, is substantially less than that observed in

samples heated to 250°C and indicates minor degradation of BZ. Collectively, the data fail to yield evidence of marked and systematic decrease in phaeomelanin marker concentrations during maturation at 100°C.

Supplementary Figs. 1–16

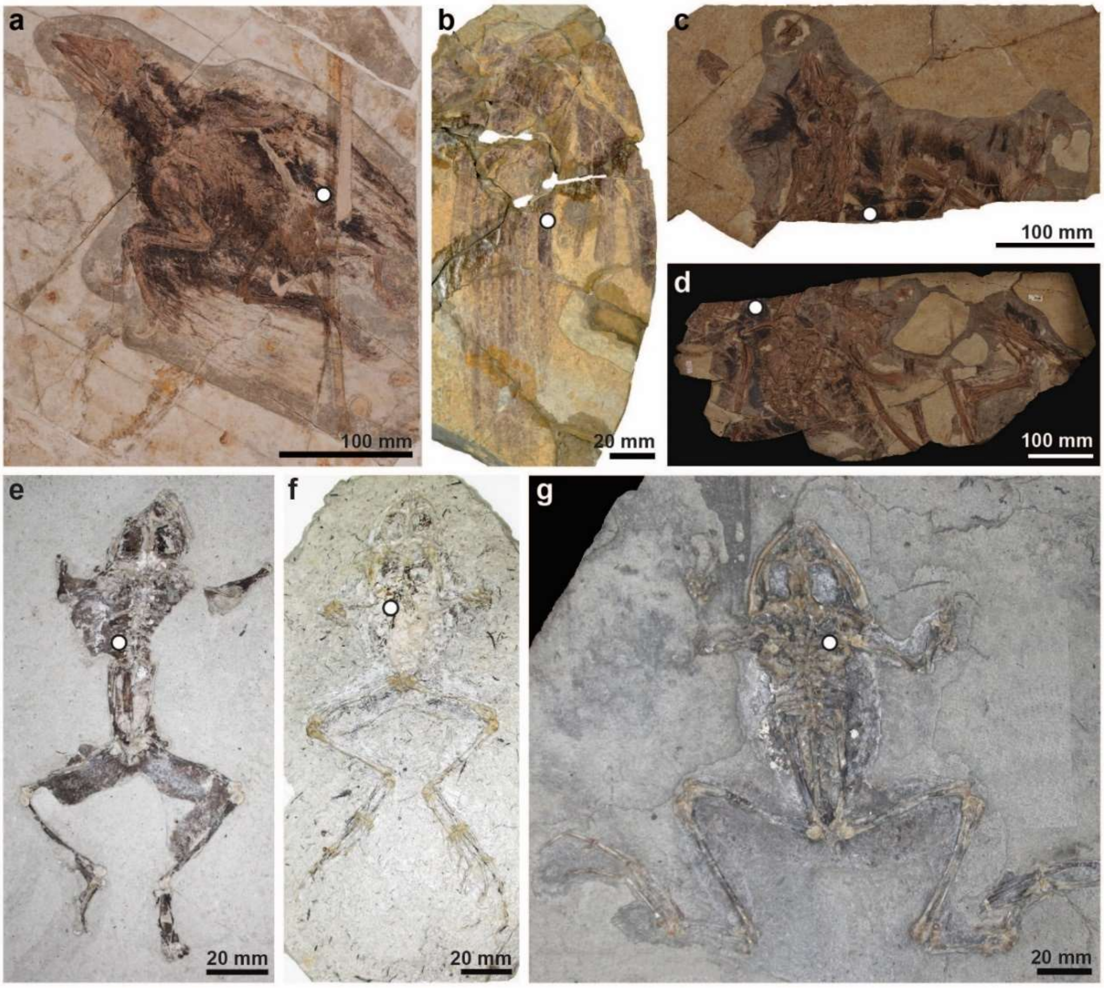

Supplementary Fig. 1 Photographs of fossil specimens used in this study. **a, b** *Confuciusornis*. **a** IVPP V 13171. **b** CKGM F 6433. **c, d** *Sinornithosaurus* (IVPP V 12811). **e–g** *Pelophylax pueyoi*. **e** MNCN 63678. **f** MNCN 63773. **g** MNCN 63694. Scale bars: **a** 100 mm **b** 20 mm **c, d** 100 mm **e–g** 20 mm. **a, c, d** from McNamara *et al.*[1]. **e, g** from McNamara *et al.*[2]. White circles indicate sampling locations.

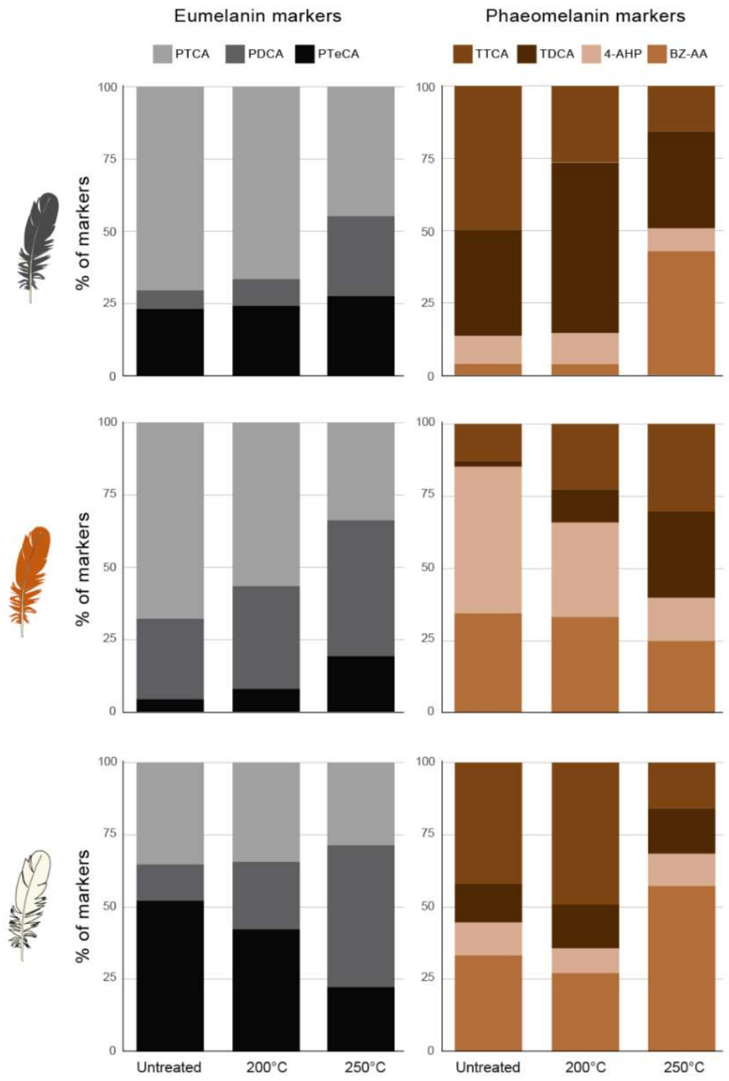

Supplementary Fig. 2 AHPO- and HI-HPLC data for black, rufous and white feathers from extant birds with, and without, thermal treatment. Eumelanin markers and the phaeomelanin markers TTCA and TDCA are derived during chemical degradation via AHPO. BZ-AA and 4-AHP are derived during HI hydrolysis. Black and rufous feathers are from the domestic chicken (*Gallus gallus*) and white feathers are from the little egret (*Egretta garzetta*). Abbreviations: PTCA, pyrrole-2,3,5-tricarboxylic acid; PDCA, pyrrole-2,3-dicarboxylic acid; PTeCA, pyrrole-2,3,4,5-tetracarboxylic acid; TTCA, thiazole-2,4,5-tricarboxylic acid; TDCA, thiazole-4,5-dicarboxylic acid; 4-AHP, 4-amino-3-hydroxyphenylalanine; BZ-AA, benzothiazole amino acid. Source data are provided as a Source Data file.

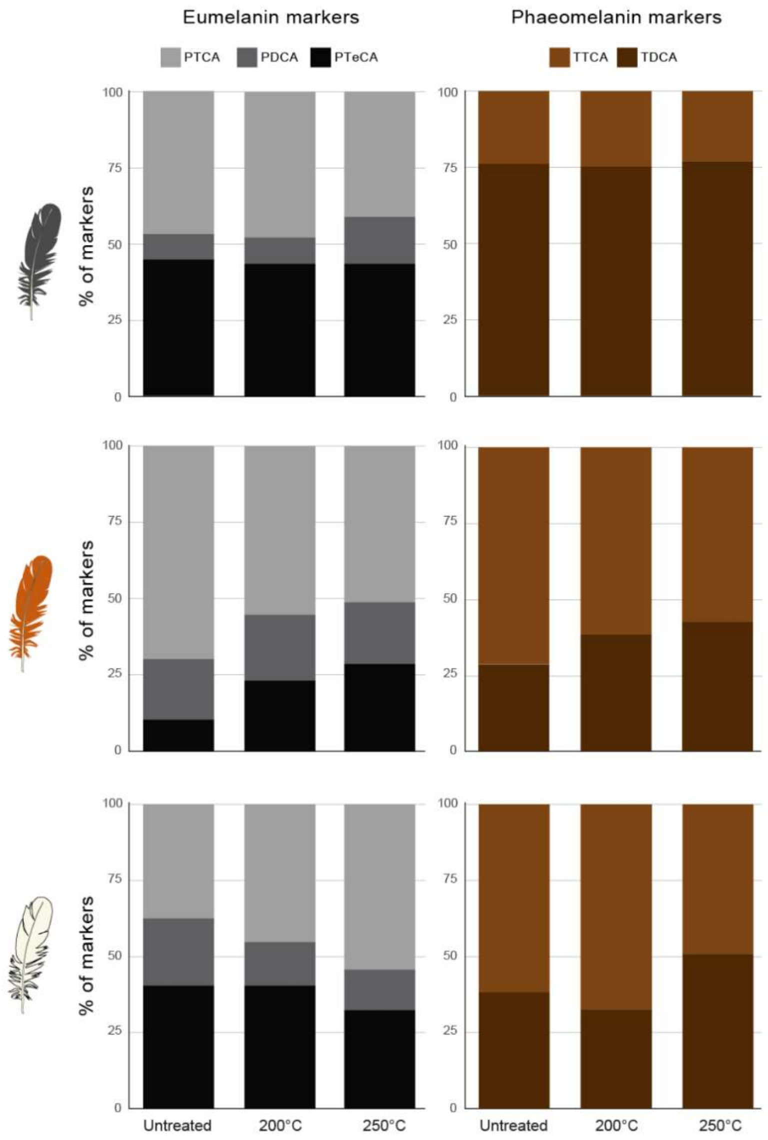

Supplementary Fig. 3 HCl-AHPO-HPLC data for black, rufous and white feathers from extant birds with, and without, thermal treatment. Melanin markers are derived during chemical degradation via HCl-AHPO. Black and rufous feathers are from the domestic chicken (*Gallus gallus*) and white feathers are from the little egret (*Egretta garzetta*). BZ and 4-AHP data are not provided as these markers cannot be analysed following acid treatment. Abbreviations: PTCA, pyrrole-2,3,5-tricarboxylic acid; PDCA, pyrrole-2,3-dicarboxylic acid; PTeCA, pyrrole-2,3,4,5-tetracarboxylic acid; TTCA, thiazole-2,4,5-tricarboxylic acid; TDCA, thiazole-4,5-dicarboxylic acid. Source data are provided as a Source Data file.

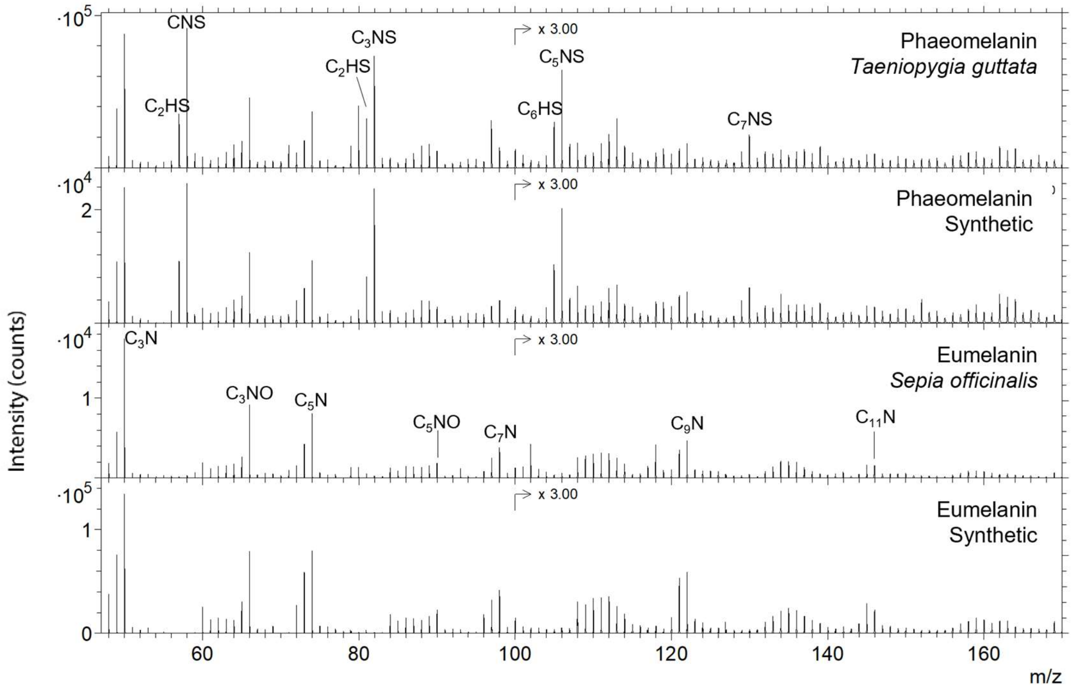

Supplementary Fig. 4 ToF-SIMS spectra for natural and synthetic phaeomelanin and eumelanin. Natural eumelanin is from the cuttlefish (*Sepia officinalis*) and natural phaeomelanin is from a melanin extract of rufous contour zebra finch (*Taeniopygia guttata*) feathers.

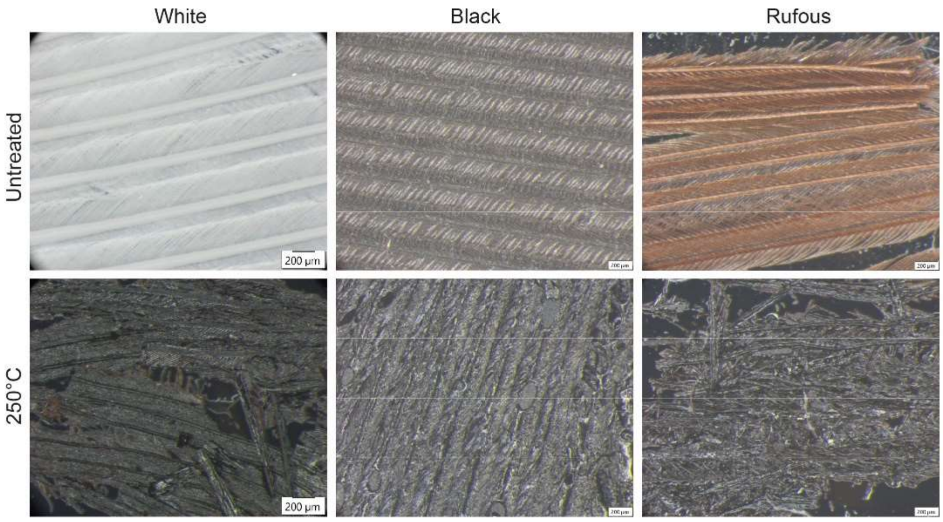

Supplementary Fig. 5 Images of untreated and experimentally treated white, black and rufous feathers. Black and rufous feathers are from the domestic chicken (*Gallus gallus*) and white feathers are from the little egret (*Egretta garzetta*). Experimentally treated feathers were matured at 250°C for 1 h.

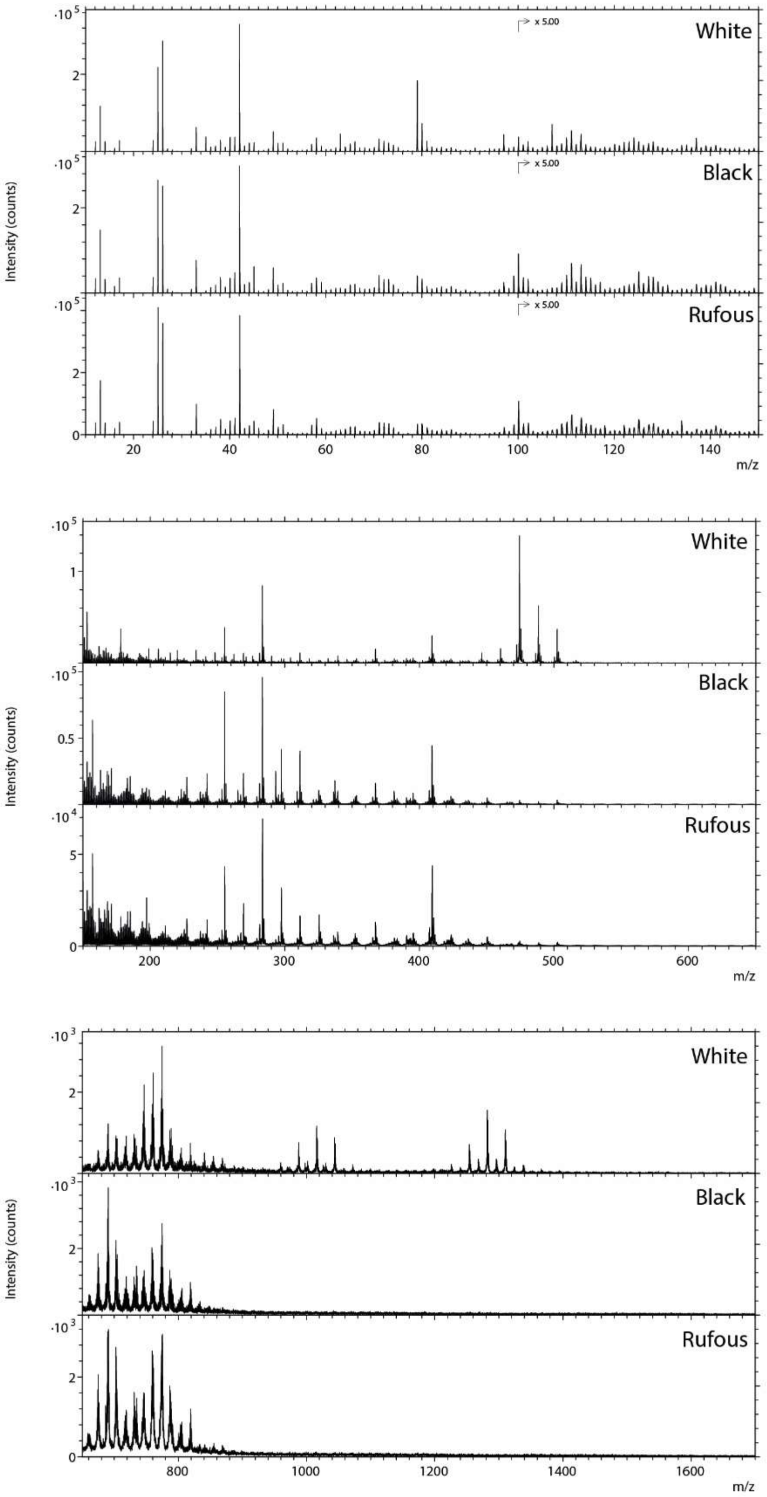

Supplementary Fig. 6 Negative ion ToF-SIMS spectra for untreated black, rufous and white feathers from extant birds. Black and rufous feathers are from the domestic chicken (*Gallus gallus*) and white feathers are from the little egret (*Egretta garzetta*).

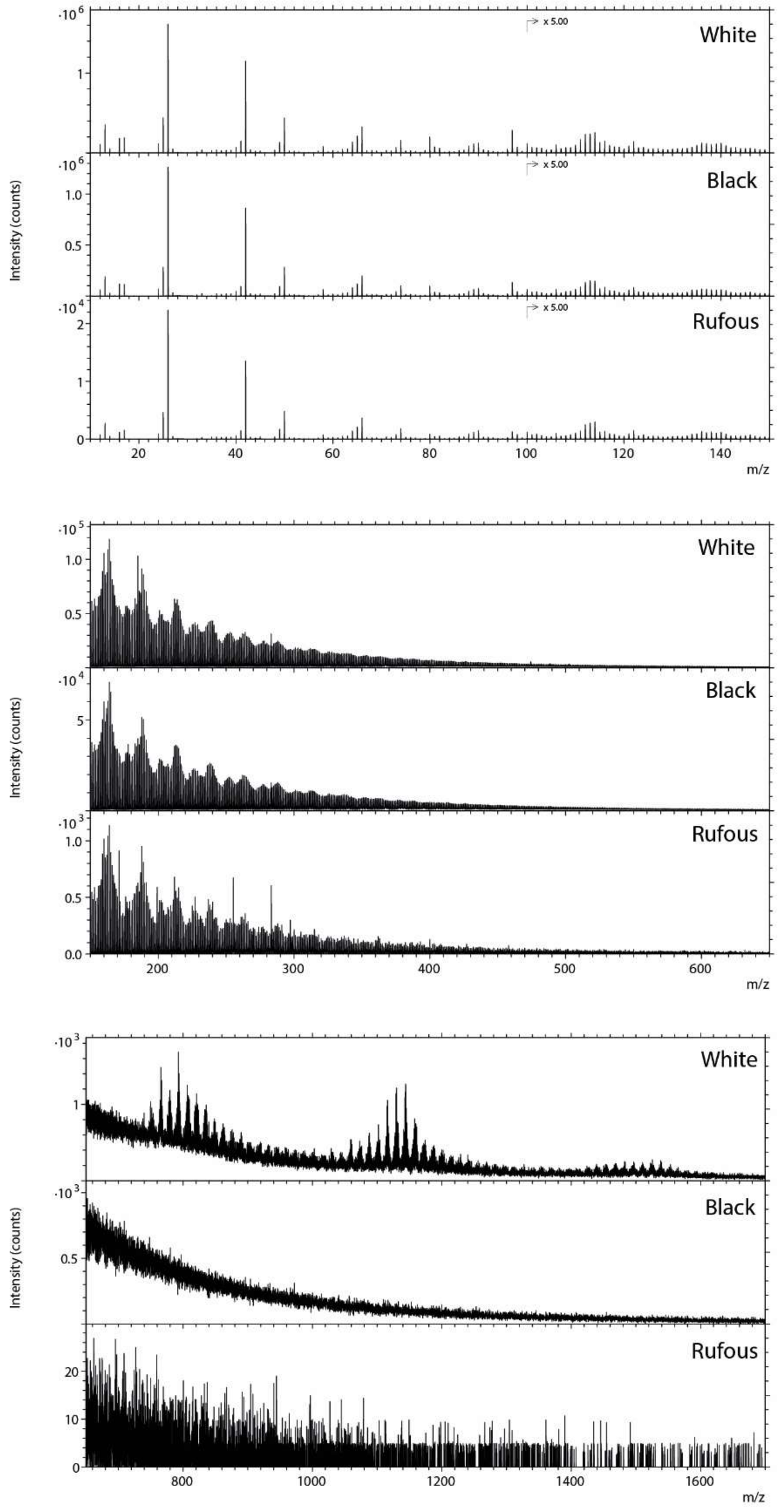

Supplementary Fig. 7 Negative ion ToF-SIMS spectra for black, rufous and white feathers from extant birds matured at 250°C for 1 h. Black and rufous feathers are from the domestic chicken (*Gallus gallus*) and white feathers are from the little egret (*Egretta garzetta*).

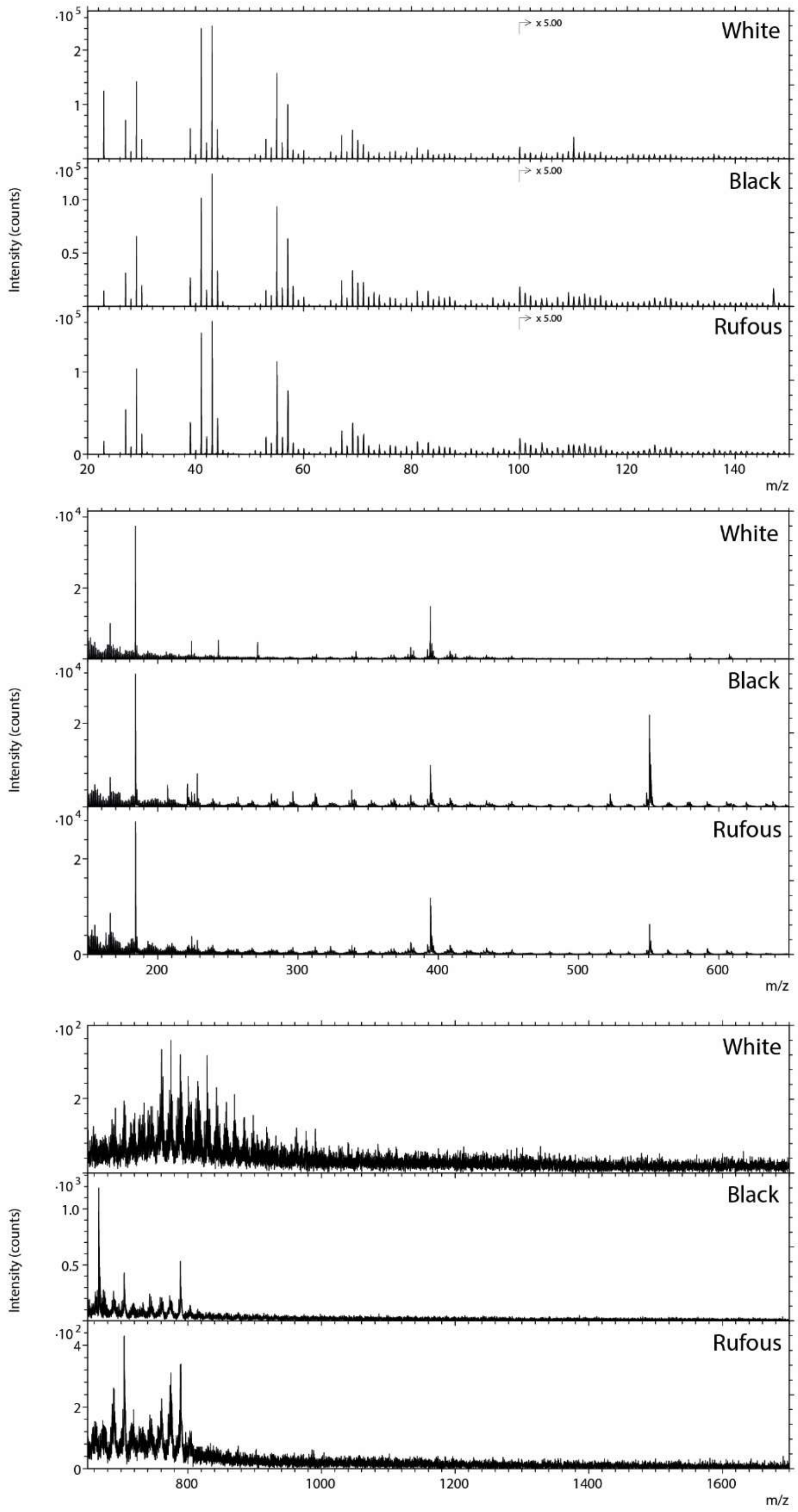

Supplementary Fig. 8 Positive ion ToF-SIMS spectra for untreated black, rufous and white feathers from extant birds. Black and rufous feathers are from the domestic chicken (*Gallus gallus*) and white feathers are from the little egret (*Egretta garzetta*).

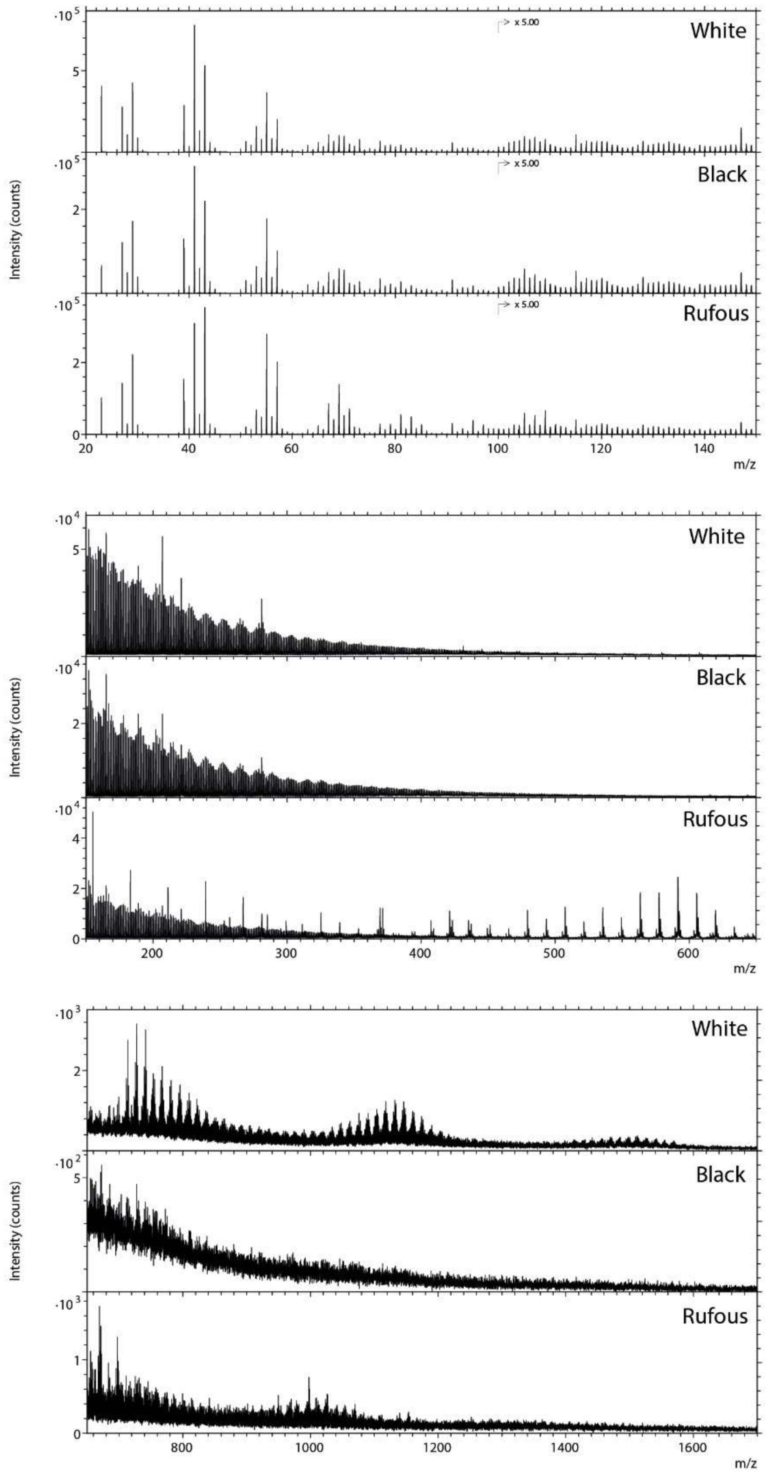

Supplementary Fig. 9 Positive ion ToF-SIMS spectra for black, rufous and white feathers from extant birds matured at 250°C for 1 h. Black and rufous feathers are from the domestic chicken (*Gallus gallus*) and white feathers are from the little egret (*Egretta garzetta*).

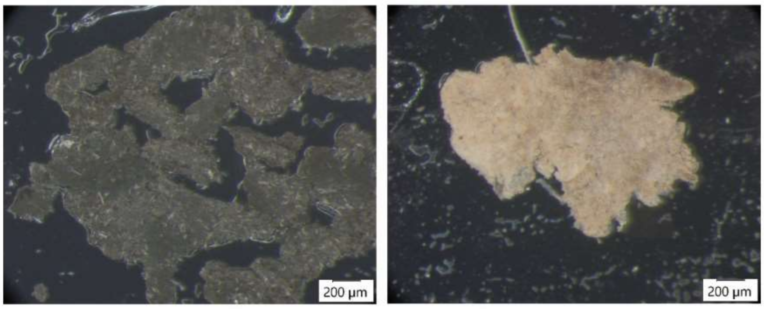

Supplementary Fig. 10 Images of untreated melanin extracts from black and rufous feathers. Eumelanin and phaeomelanin extracts are from black (left) and rufous (right) zebra finch (*Taeniopygia guttata*) feathers, respectively.

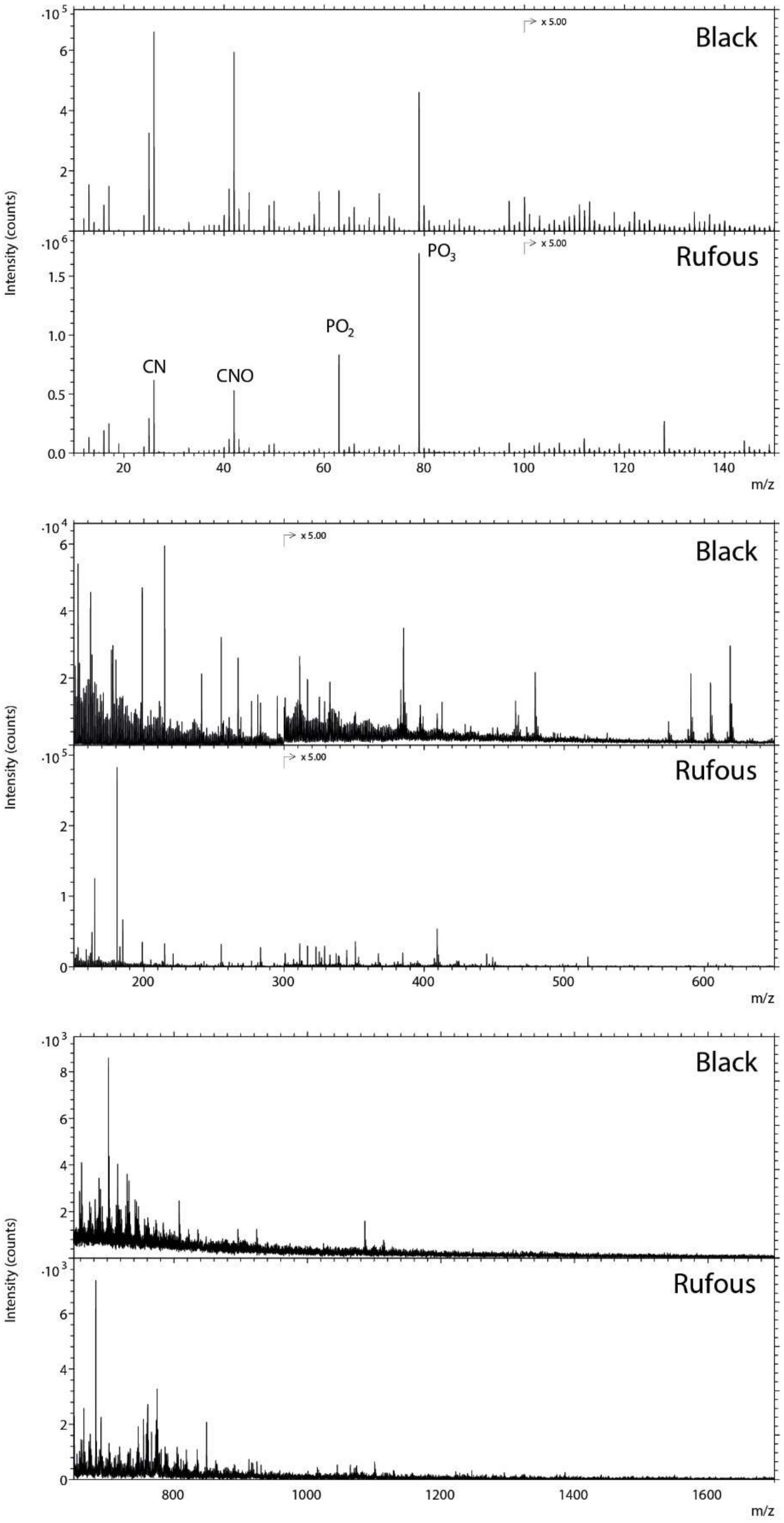

Supplementary Fig. 11 Negative ion ToF-SIMS spectra for melanin extracts from untreated black and rufous feathers. Black and rufous feathers are from the zebra finch (*Taeniopygia guttata*). See Fig. S10 for images of melanin extracts.

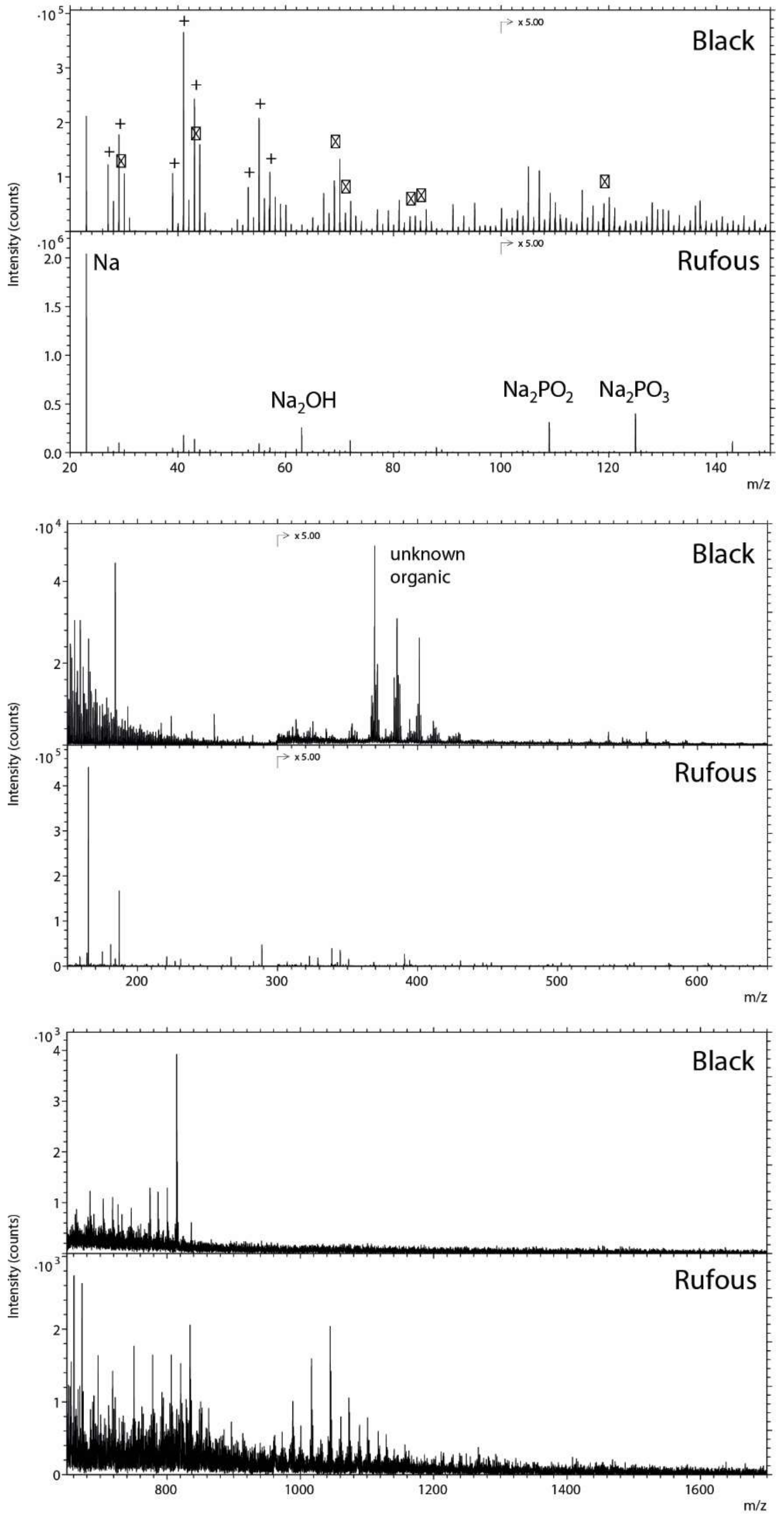

Supplementary Fig. 12 Positive ion ToF-SIMS spectra for melanin extracts from untreated black and rufous feathers. Black and rufous feathers are from the zebra finch (*Taeniopygia guttata*). See Fig. S10 for images of melanin extracts.

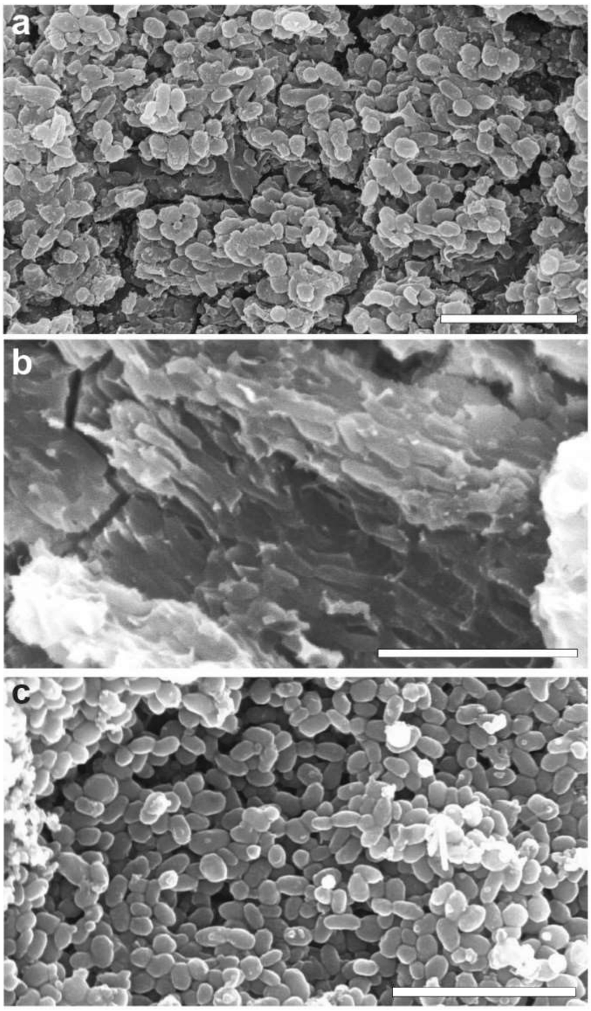

Supplementary Fig. 13 Scanning electron micrographs of fossil melanosomes in fossil specimens. **a** *Confuciusornis* (IVPP V 13171). **b** *Sinornithosaurus* (IVPP V 12811). **c** *Pelophylax pueyoi* (MNCN 63805), modified from McNamara *et al.*[3]. Scale bars, 5 μm.

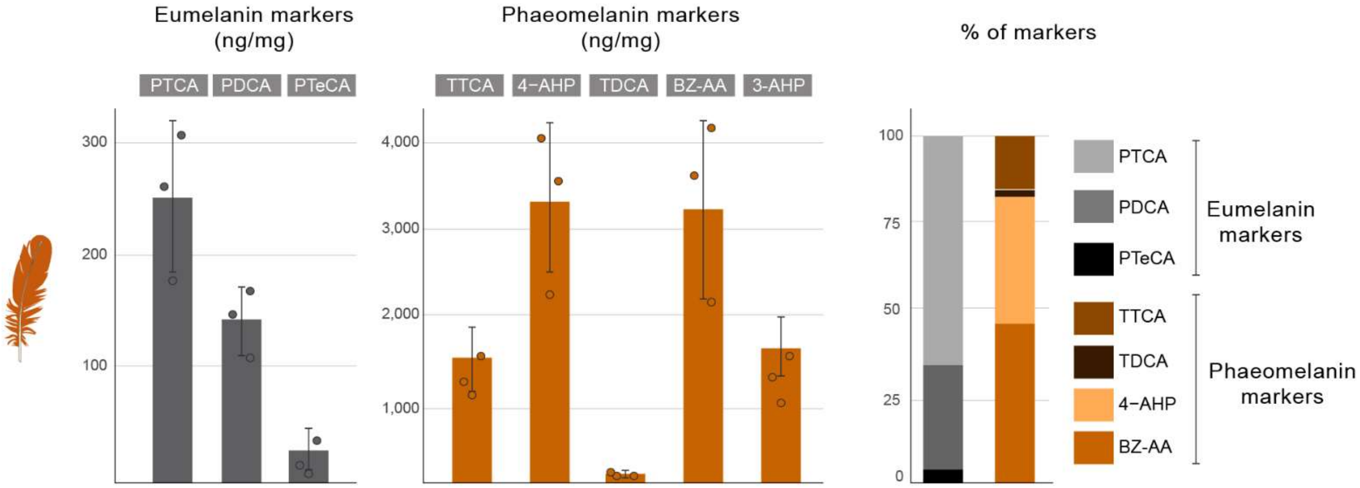

Supplementary Fig. 14 AHPO- and HI-HPLC data for rufous feathers from the domestic chicken (*Gallus gallus*) matured at 100°C. Eumelanin markers and the phaeomelanin markers TTCA and TDCA are derived during chemical degradation via AHPO of rufous feathers (n = 3). BZ-AA and 4-AHP are derived during HI hydrolysis. Data in ng/mg are presented as mean ± SD. Abbreviations: PTCA, pyrrole-2,3,5-tricarboxylic acid; PDCA, pyrrole-2,3-dicarboxylic acid; PTeCA, pyrrole-2,3,4,5-tetracarboxylic acid; TTCA, thiazole-2,4,5-tricarboxylic acid; 4-AHP, 4-amino-3-hydroxyphenylalanine; TDCA, thiazole-4,5-dicarboxylic acid; BZ-AA, benzothiazole amino acid; 3-AHP, 3-amino-4-hydroxyphenylalanine. Source data are provided as a Source Data file.

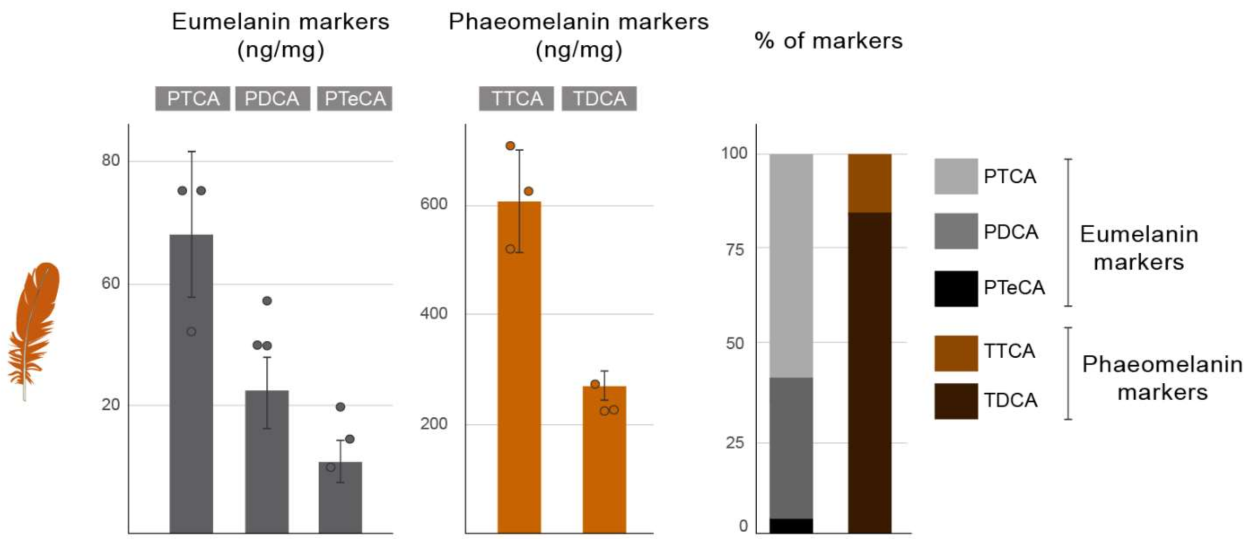

Supplementary Fig. 15 HCl-AHPO-HPLC data for rufous feathers from the domestic chicken (*Gallus gallus*) matured at 100°C. Melanin markers are derived during chemical degradation via HCl-AHPO of rufous feathers (n = 3). BZ and 4-AHP data are not provided as these markers cannot be analysed following acid treatment. Data in ng/mg are presented as mean ± SD. Abbreviations: PTCA, pyrrole-2,3,5-tricarboxylic acid; PDCA, pyrrole-2,3-dicarboxylic acid; PTeCA, pyrrole-2,3,4,5-tetracarboxylic acid; TTCA, thiazole-2,4,5-tricarboxylic acid; TDCA, thiazole-4,5-dicarboxylic acid. Source data are provided as a Source Data file.

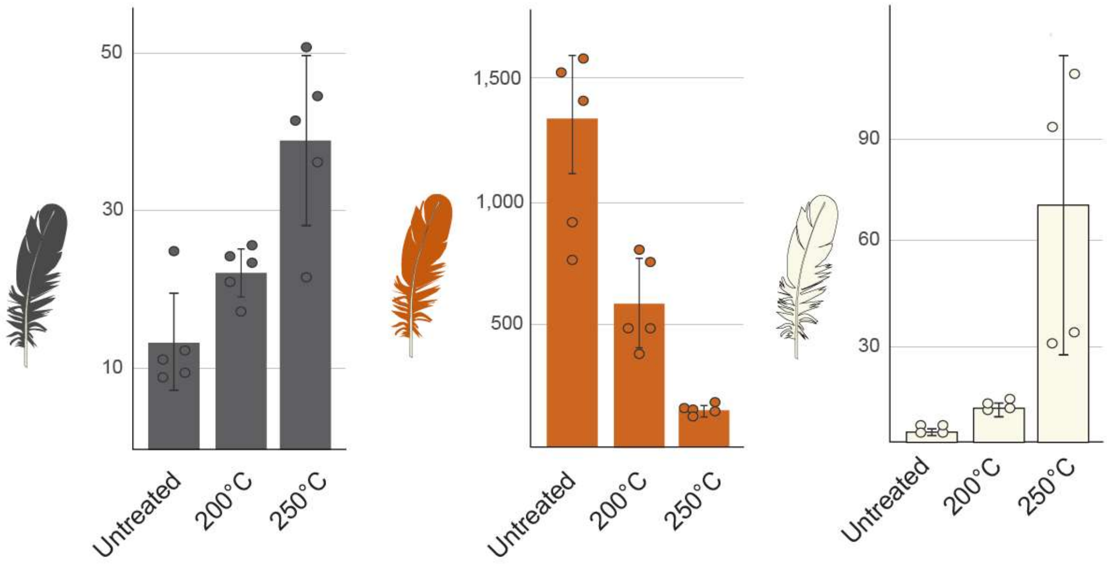

Supplementary Fig. 16 HI-HPLC data for the phaeomelanin marker 3-AHP (3-amino-4-hydroxyphenylalanine) for black, rufous and white feathers from extant birds with, and without, thermal treatment. Black and rufous feathers are from the domestic chicken (each n = 5; *Gallus gallus*) and white feathers are from the little egret (n = 4; *Egretta garzetta*). The unit of the vertical axis in the figure is ng/mg. Data are presented as mean ± SD. Source data are provided as a Source Data file.

Supplementary Table 1. List of study samples and analyses performed on each.

			Analysis				
Feather colour	Species	Treatment	AHPO	HCl-AHPO	HI hydrolysis	Soluene-350 solubilisation	ToF-SIMS
Black	*Gallus gallus*	Untreated	X	X	X	X	X
Black	*Gallus gallus*	Untreated	X	X	X	X	-
Black	*Gallus gallus*	Untreated	X	X	X	X	-
Black	*Gallus gallus*	Untreated	X	X	X	-	-
Black	*Gallus gallus*	Untreated	X	X	X	-	-
Black	*Gallus gallus*	200°C	X	X	X	-	-
Black	*Gallus gallus*	200°C	X	X	X	-	-
Black	*Gallus gallus*	200°C	X	X	X	-	-
Black	*Gallus gallus*	200°C	X	X	X	-	-
Black	*Gallus gallus*	200°C	X	X	X	-	-
Black	*Gallus gallus*	250°C	X	X	X	-	X
Black	*Gallus gallus*	250°C	X	X	X	-	-
Black	*Gallus gallus*	250°C	X	X	X	-	-
Black	*Gallus gallus*	250°C	X	X	X	-	-
Black	*Gallus gallus*	250°C	X	X	X	-	-
Rufous	*Gallus gallus*	Untreated	X	X	X	X	X
Rufous	*Gallus gallus*	Untreated	X	X	X	X	-
Rufous	*Gallus gallus*	Untreated	X	X	X	X	-
Rufous	*Gallus gallus*	Untreated	X	X	X	-	-
Rufous	*Gallus gallus*	Untreated	X	X	X	-	-
Rufous	*Gallus gallus*	100°C	X	X	X	-	-
Rufous	*Gallus gallus*	100°C	X	X	X	-	-
Rufous	*Gallus gallus*	100°C	X	X	X	-	-
Rufous	*Gallus gallus*	200°C	X	X	X	-	-
Rufous	*Gallus gallus*	200°C	X	X	X	-	-
Rufous	*Gallus gallus*	200°C	X	X	X	-	-
Rufous	*Gallus gallus*	200°C	X	X	X	-	-
Rufous	*Gallus gallus*	200°C	X	X	X	-	-
Rufous	*Gallus gallus*	250°C	X	X	X	-	X
Rufous	*Gallus gallus*	250°C	X	X	X	-	-
Rufous	*Gallus gallus*	250°C	X	X	X	-	-
Rufous	*Gallus gallus*	250°C	X	X	X	-	-
Rufous	*Gallus gallus*	250°C	X	X	X	-	-
White	*Egretta garzetta*	Untreated	X	X	X	X	X
White	*Egretta garzetta*	Untreated	X	X	X	X	-
White	*Egretta garzetta*	Untreated	X	X	X	X	-
White	*Egretta garzetta*	Untreated	X	X	X	-	-
White	*Egretta garzetta*	200°C	X	X	X	-	-
White	*Egretta garzetta*	200°C	X	X	X	-	-
White	*Egretta garzetta*	200°C	X	X	X	-	-
White	*Egretta garzetta*	200°C	X	X	X	-	-
White	*Egretta garzetta*	250°C	X	X	X	-	X
White	*Egretta garzetta*	250°C	X	X	X	-	-
White	*Egretta garzetta*	250°C	X	X	X	-	-
White	*Egretta garzetta*	250°C	X	X	X	-	-
Black	*Taeniopygia guttata*	Untreated melanin extract	-	-	-	-	X
Rufous	*Taeniopygia guttata*	Untreated melanin extract	-	-	-	-	X

Supplementary Table 2. HPLC-AHPO data for individual melanin marker concentrations (ng/mg) for supplementary maturation experiments. Experiments used rufous *Gallus gallus* feathers (n = 3 for experiments with, and without, HCl treatment). Abbreviations: PTCA, pyrrole-2,3,5-tricarboxylic acid; PDCA, pyrrole-2,3-dicarboxylic acid; PTeCA, pyrrole-2,3,4,5-tetracarboxylic acid; TTCA, thiazole-2,4,5-tricarboxylic acid; TDCA, thiazole-4,5-dicarboxylic acid; BZ-AA, benzothiazole amino acid; 4-AHP, 4-amino-3-hydroxyphenylalanine; 3-AHP, 3-amino-4-hydroxyphenylalanine. Source data are provided as a Source Data file.

			Eumelanin markers			Phaeomelanin markers				
Feather type		HCl pre-treatment	PTCA	PDCA	PTeCA	TTCA	TDCA	BZ-AA	4-AHP	3-AHP
Rufous	100°C	no	249.33 ± 66.91	142.33 ± 29.28	23.33 ± 15.4	1394.67 ± 257.47	3352.33 ± 1056.81	1.6 ± 0.96	3319.67 ± 937.05	1392.67 ± 306.78
		yes	65.23 ± 19.08	29.27 ± 6.87	15.47 ± 4.43	607.67 ± 95.21	243.67 ± 28.11	X	X	X

References

1 McNamara, M. E. *et al.* Fossilized skin reveals coevolution with feathers and metabolism in feathered dinosaurs and early birds. *Nat. Commun.* **9,** 2072 (2018).

2 McNamara, M. E., Orr, P. J., Alcalá, L., Anadón, P. & Peñalver, E. What controls the taphonomy of exceptionally preserved taxa—environment or biology? A case study using frogs from the Miocene Libros Konservat-Lagerstätte (Teruel, Spain). *Palaios* **27,** 63–77 (2012).

3 McNamara, M. E. *et al.* Soft-tissue preservation in Miocene frogs from Libros, Spain: insights into the genesis of decay microenvironments. *Palaios* **24,** 104–117 (2009).
