# Supplementary material for: Taphonomic experiments reveal authentic molecular signals for fossil melanins and verify preservation of phaeomelanin in fossils
Source: Nat Commun. 2023 Oct 6;14:5651. doi: 10.1038/s41467-023-40570-w (PMC10558522; doi:10.1038/s41467-023-40570-w)
Supplement: Supplementary file 2 — Reporting Summary [file 41467_2023_40570_MOESM2_ESM.pdf]

nature portfolio

Corresponding author(s): Tiffany S. Slater and Maria E. McNamara

Last updated by author(s): Jul 18, 2023

Reporting Summary

Nature Portfolio wishes to improve the reproducibility of the work that we publish. This form provides structure for consistency and transparency in reporting. For further information on Nature Portfolio policies, see our Editorial Policies and the Editorial Policy Checklist.

Statistics

For all statistical analyses, confirm that the following items are present in the figure legend, table legend, main text, or Methods section.

n/a	Confirmed	
☐	☒	The exact sample size (*n*) for each experimental group/condition, given as a discrete number and unit of measurement
☐	☒	A statement on whether measurements were taken from distinct samples or whether the same sample was measured repeatedly
☒	☐	The statistical test(s) used AND whether they are one- or two-sided<br>*Only common tests should be described solely by name; describe more complex techniques in the Methods section.*
☒	☐	A description of all covariates tested
☒	☐	A description of any assumptions or corrections, such as tests of normality and adjustment for multiple comparisons
☐	☒	A full description of the statistical parameters including central tendency (e.g. means) or other basic estimates (e.g. regression coefficient) AND variation (e.g. standard deviation) or associated estimates of uncertainty (e.g. confidence intervals)
☒	☐	For null hypothesis testing, the test statistic (e.g. *F*, *t*, *r*) with confidence intervals, effect sizes, degrees of freedom and *P* value noted<br>*Give P values as exact values whenever suitable.*
☒	☐	For Bayesian analysis, information on the choice of priors and Markov chain Monte Carlo settings
☒	☐	For hierarchical and complex designs, identification of the appropriate level for tests and full reporting of outcomes
☒	☐	Estimates of effect sizes (e.g. Cohen's *d*, Pearson's *r*), indicating how they were calculated

Our web collection on statistics for biologists contains articles on many of the points above.

Software and code

Policy information about availability of computer code

Data collection	No software was used for the completion of this study.
Data analysis	No software was used for the completion of this study.

For manuscripts utilizing custom algorithms or software that are central to the research but not yet described in published literature, software must be made available to editors and reviewers. We strongly encourage code deposition in a community repository (e.g. GitHub). See the Nature Portfolio guidelines for submitting code & software for further information.

Data

Policy information about availability of data

All manuscripts must include a data availability statement. This statement should provide the following information, where applicable:

- Accession codes, unique identifiers, or web links for publicly available datasets
- A description of any restrictions on data availability
- For clinical datasets or third party data, please ensure that the statement adheres to our policy

The HPLC data generated in this study are provided in the Source Data file.

Human research participants

Policy information about studies involving human research participants and Sex and Gender in Research.

Reporting on sex and gender	N/A
Population characteristics	N/A
Recruitment	N/A
Ethics oversight	N/A

Note that full information on the approval of the study protocol must also be provided in the manuscript.

Field-specific reporting

Please select the one below that is the best fit for your research. If you are not sure, read the appropriate sections before making your selection.

☐ Life sciences ☐ Behavioural & social sciences ☒ Ecological, evolutionary & environmental sciences

For a reference copy of the document with all sections, see nature.com/documents/nr-reporting-summary-flat.pdf

Ecological, evolutionary & environmental sciences study design

All studies must disclose on these points even when the disclosure is negative.

Study description	Black (n = 5) and rufous (n = 5) contour feathers from Gallus gallus domesticus and white (n = 4) primary feathers from Egretta garzetta were each sampled (ca. 45 x 25 mm) and left untreated or matured at 200°C or 250°C. Additional rufous (n = 3) contour feathers from Gallus gallus domesticus were each sampled (ca. 45 x 25 mm) and matured at 100°C. 3-5 mg of each untreated and experimentally treated sample were subjected to alkaline hydrogen peroxide oxidation-high-performance liquid chromatography or hydroiodic acid hydrolysis. Synthetic eumelanin and phaeomelanin, cuttlefish (Sepia officinalis) melanin, experimentally treated feather samples and melanin extracts from untreated black (n = 1) and rufous (n = 2) contour zebrafinch feathers (Taeniopygia guttata) were analzed using ToF-SIMS.
Research sample	Black (n = 5) and rufous (n = 5) contour feathers from Gallus gallus domesticus and white (n = 4) primary feathers from Egretta garzetta comprised the research sample for untreated feathers and feathers matured at 200°C and 250°C. The black and rufous feathers represent tissues that contain predominantly eumelanin and phaeomelanin, respectively; white feathers represent tissues that lack melanin. Black feathers from Gallus gallus domesticus were chosen as preliminary analyses revealed the presence of eumelanin in very high quantities compared to other common birds (Eurasian magpie, Pica pica; wood pigeon, Columba palumbus; raven, Corvus corax; crow, Corvus sp.) with black feathers. Rufous feathers from Gallus gallus domesticus were chosen based on a visual assessment of their rufous colouration and the readily available nature of the material. White feathers from Egretta garzetta were chosen due to the (near-) absence of melanin and the readily available nature of the material. Additional rufous (n = 3) contour feathers from Gallus gallus domesticus comprised the research sample for feathers matured at 100°C. Synthetic eumelanin and phaeomelanin, cuttlefish (Sepia officinalis) melanin and melanin extracts from untreated black (n = 1) and rufous (n = 2) contour zebrafinch feathers (Taeniopygia guttata) were used to analyse pure eumelanin and phaeomelanin in its natural and synthetic forms.
Sampling strategy	No sample size calculation was performed. Five samples were analyzed for each of black and orange feathers (and four samples of white feathers), per experimental treatment, in order to assess reproducibility. Analysis of additional samples was not feasible due to limited material and/or temporal constraints.
Data collection	HPLC data were collected by Prof. Shosuke Ito and Prof. Kazumasa Wakamatsu using a JASCO 880-PU pump (JASCO Co., Tokyo, Japan), a C18 column (Capcell Pak MG; 4.6 × 250 mm; 5 µm particle size, Osaka Soda, Osaka, Japan) and a JASCO UV detector (JASCO Co., Tokyo, Japan). Spectra were obtained on 07.02.2020 (AHPO and HCl-AHPO data excluding BZ), 09.07.2020 (BZ data), 18.09.2020 and 21.10.2021 (fossil data) and 30.09.2021 (AHPO and HCl-AHPO additional replicates) and peaks were quantified, with values recorded in an excel spreadsheet. ToF-SIMS spectra were collected by Dr Johan Lindgren, Dr Martin Jarenmark and Dr Peter Sjovall using a TOFSIMS IV instrument (IONTOF GmbH).
Timing and spatial scale	Data were not collected with a time or spatial scale.
Data exclusions	No data were excluded from the analyses.
Reproducibility	No attempts were made to repeat this study.
Randomization	Randomization was not relevant to our study as there were no opportunities for bias.
Blinding	Blinding was not relevant to our study as qualitative data were obtained and interpreted.

Did the study involve field work? ☐ Yes ☒ No

Reporting for specific materials, systems and methods

We require information from authors about some types of materials, experimental systems and methods used in many studies. Here, indicate whether each material, system or method listed is relevant to your study. If you are not sure if a list item applies to your research, read the appropriate section before selecting a response.

Materials & experimental systems

n/a	Involved in the study
☒	☐ Antibodies
☒	☐ Eukaryotic cell lines
☐	☒ Palaeontology and archaeology
☐	☒ Animals and other organisms
☒	☐ Clinical data
☒	☐ Dual use research of concern

Methods

n/a	Involved in the study
☒	☐ ChIP-seq
☒	☐ Flow cytometry
☒	☐ MRI-based neuroimaging

Palaeontology and Archaeology

Specimen provenance	Fossil specimens were not recovered in the field but are from collections of recognized public institutions. Permission for destructive sampling was granted by the museum curators. No permits were required by the institutions for this study.
Specimen deposition	CKGM, Cork Geological Museum; IVPP, Institute of Vertebrate Paleontology and Paleoanthropology, Beijing, China; MNCN, Museu Nacional de Ciencias Naturales, Madrid, Spain.
Dating methods	No new dates are provided for fossil specimens in this study.

☐ Tick this box to confirm that the raw and calibrated dates are available in the paper or in Supplementary Information.

Ethics oversight	No ethical approval or guidance was required as specimens were not recovered in the field.

Note that full information on the approval of the study protocol must also be provided in the manuscript.

Animals and other research organisms

Policy information about studies involving animals; ARRIVE guidelines recommended for reporting animal research, and Sex and Gender in Research

Laboratory animals	This study did not involve laboratory animals.
Wild animals	The wild little egret used in this study was deceased when found.
Reporting on sex	Information on sex could not be recorded for all samples as the Gallus gallus domesticus feather samples were from shed feathers that were donated by private individuals (hen-owners).
Field-collected samples	The wild little egret used in this study was deceased when found and collected in an urban area of Cork, Ireland.
Ethics oversight	No ethical approval was required for this study. Gallus gallus domesticus feather samples were from shed feathers donated by private individuals (hen-owners) and the little egret was deceased when found. Zebrafinch were purchased from animal suppliers for previously published studies. Euthanasia of zebrafinch for previous studies was either approved by the Health Products Regulatory Authority of Ireland via authorization AE19130-IO87 for black and rufous feathers (each n = 1) or performed according to Swedish regulations for a rufous feather (n = 1). All zebrafinch feather samples obtained were used for enzymatic melanin extraction and subsequent analysis using ToF-SIMS.

Note that full information on the approval of the study protocol must also be provided in the manuscript.
